# Distinct Communities and Differing Dispersal Routes in Bacteria and Fungi of Honey Bees, Honey, and Flowers

**DOI:** 10.1007/s00248-024-02413-z

**Published:** 2024-07-30

**Authors:** Mikko Tiusanen, Antoine Becker-Scarpitta, Helena Wirta

**Affiliations:** 1 Department of Agricultural Sciences, University of Helsinki, Helsinki, Finland; 2 Department of Environmental Systems Science, Eidgenössische Technische Hochschule Zürich (ETH Zürich), Zurich, Switzerland; 3 UMR PVBMT, CIRAD, Saint Pierre, 97410 La Réunion, France; 4 Department of Ecology and Environmental Science, Umeå University, Umeå, Sweden

**Keywords:** Pollination, *Apis mellifera*, Microbe, DNA metabarcoding

## Abstract

Microbiota, the communities of microbes on and in organisms or organic matter, are essential for the functioning of ecosystems. How microbes are shared and transmitted delineates the formation of a microbiota. As pollinators forage, they offer a route to transfer microbes among the flowering plants, themselves, and their nests. To assess how the two components of the microbiota, bacteria and fungi, in pollination communities are shared and transferred, we focused on the honey bee *Apis mellifera* and collected honey bee, honey (representing the hive microbiota), and flower samples three times during the summer in Finland. We identified the bacteria and fungi by DNA metabarcoding. To determine the impact of honey bees’ flower choices on the honey bee and hive microbiota, we identified also plant DNA in honey. The bacterial communities of honey bees, honey, and flowers all differ greatly from each other, while the fungal communities of honey bees and honey are very similar, yet different from flowers. The time of the summer and the sampling area influence all these microbiota. For flowers, the plant identity impacts both bacterial and fungal communities’ composition the most. For the dispersal pathways of bacteria to honey bees, they are acquired directly from the honey and indirectly from flowers through the honey, while fungi are directly transmitted to honey bees from flowers. Overall, the distinctiveness of the microbiota of honey bees, honey, and the surrounding flowers suggests the sharing of microbes among them occurs but plays a minor role for the established microbiota.

**Supplementary Information:**

The online version contains supplementary material available at 10.1007/s00248-024-02413-z.

## Introduction

We are surrounded by communities of microbes living on and in organisms or organic matter. This microbiota is needed for the functioning of ecosystems and the health of organisms [1]. How microbes are transmitted among organisms is crucial in the formation of an individual’s microbiota [2]. Overall, organisms acquire microbiota in different ways. As an example, as a honey bee emerges, it has no gut microbes, but within a few days, they are obtained through contact with nurse bees and nurse bees’ feces [3]. In general, the microbiota of an individual is acquired through three modes: maternal inheritance, social transmission, and environmental acquisition [4]. All of these modes impact the microbiota of insects [2, 5]. In addition, suitability of the host as a habitat for the microbe, the host filtering of microbes, and the microbe–microbe interactions within the host determine the composition of a microbiota [6].

Pollinators play an irreplaceable ecological role, as the vast majority of flowering plants need animal-mediated pollination to ensure the quantity and quality of fruits and seeds [7, 8]. In regard to many important crop species, honey bees contribute significantly to their pollination [9]. While foraging and pollinating, pollinators contribute to the dispersal of microbes in and on plants [10], and microbes can be seen as the third partners of bee-angiosperm mutualism [4]. Microbes may inhabit flower tissues, such as pollen, ovules, and seeds, or reside on the surfaces of the flower organs or in nectar. For instance, the microbes in nectar are dispersal-limited, making pollinators crucial vectors for them [11]. Bacteria and fungi living on flowers and in nectar can alter a flower’s attractiveness to pollinators [7, 12]. Conversely, the identity of the pollinator and its nectar-foraging behavior can influence the nectar’s microbial community [13]. Overall, microbes and their chemical effects can influence the interactions between pollinators and flowers and thus affect pollination [7, 14]. However, understanding the structures of the microbiota within pollination communities remains fragmented [15].

Honey bees, *Apis mellifera*, are the most abundant pollinators of the world [8, 16]. Honey bees harbor a distinct, well-studied gut microbiota, mainly consisting of bacteria [17, 18], and being mainly transmitted by social interactions among colony members, and to a lesser extent by hive surfaces [19, 20]. Also, the environment shapes the gut bacterial community of honey bees [21, 22]. On the other hand, honey bees influence the microbiota of flowers [21], and the environment influences the overall microbiota of honey bees [22]. On top of interacting with the microbes in their guts, honey bees interact with microbes in their food stores, hive surfaces, floral resources, and other parts of their environment. There, the importance of microbial dispersal from different sources to honey bees remains unexplored [23] yet would be important because of their interplay [24]. Honey bees have several well-known pathogens [25–27]. Some of them are known to be spread to other pollinators too, possibly through flowers [28–30]. Therefore, it is important to understand the pathway of transmission. The hives and food stores of honey bees also host a diversity of microbes, with different environmental factors shaping these microbial communities [31, 32]. Flowers share microbes with the visiting bees and the food stores of honey bees [23, 33, 34], yet host physiology and priority effects affect which microbes can establish in and on a honey bee [7]. Most research on the microbes of honey bees, of their nests, and of the flowers they visit has focused on bacteria (for an exception, see a comparison of bacteria and fungi in bee bread, the stored pollen [35]), although fungi also play an important role in pollination communities by changing the attractiveness of nectar differently from bacteria [36, 37], for example, pathogenic fungi, species of *Vairimorpha* (*Nosema*), are known to be distributed through flowers to honey bees and bumble bees [38]. For another managed bee, *Megachile rotundata*, bacteria and fungi from floral resources are brought to their nests, and their nests are likely to serve as reservoirs of both bacterial and fungal pathogens of flowers and the bees themselves to act as transferring agents [39].

Given the vast role in pollination attributed to the honey bee, we examine how bacteria and fungi are shared and transferred by honey bees, among the pollinating species itself, its nest (i.e., the hive), and its food resources (i.e., flowers in the surroundings). For assessing bacteria and fungi in the hives, we sample honey, which stores DNA well. The processing of nectar into honey includes spreading it into a high number of open combs in small amounts, for excess water to evaporate [40], and this allows DNA present in any form within the hive to enter honey. The time taken for nectar to ripen into honey tends to be 3 to 7 days after which honey bees collect the nectar, now turned into honey, from multiple combs into few to fill the combs up and cover these with wax [40, 41]. Thus, the bacterial and fungal DNA in newly covered honey would offer a sample of the bacteria and fungi honeybees have encountered in their hive during approximately the previous week. While the structure of the pollination network has been pin-pointed as important for the dispersal of the microbes to flowers [23], here, we aim at identifying the movement of microbes and their dispersal routes to honey bees, through their hives and through flowers. We identify and compare the bacterial and fungal communities of honey bees, honey (representing their hives), and flowers in the surroundings of the hives by DNA metabarcoding. We focus our analyses onto the strain level (genetic variants of microbe species), as this is essential to detect which exact microbes are shared [2]. For this, we use zero-radius operational taxonomic units (ZOTUs) [42], the smallest taxonomic units based on sequence data.

We examine the following questions:

1. The honey bees, their honey, and surrounding flowers share bacteria and fungi?
2. What is the spatiotemporal structure of the bacterial and fungal communities of honey bees, honey, and flowers?
3. How do the bacteria and fungi from flowers and the honey transfer to the microbiota of the honey bees?

## Methods

### Sampling, Sample Preprocessing, and Dna Metabarcoding

To study the microbiota of honey bees, honey, and flowers, we studied 36 honey bee colonies (29 colonies at the end of the summer), placed in six apiaries [as in 43] and the surrounding flowering plants in South-Finland.

We collected honey bee, honey, and flower samples in mid-June, mid-July, and mid-August in 2021. For microbiota of honey bees, we collected one deciliter of a mixture of nurse and forager bees, and a subsample of this was homogenized to determine microbiota in and on honey bees. We used whole individuals, as the whole-body microbiota represent the gut microbiome and also capture the whole-body microbial exposure [44]. For the honey microbiota, we collected newly covered honey. For microbiota of flowers, we sampled inflorescences of the abundantly flowering species into 99% EtOH in different habitat types close to the apiaries (herbaceous plants and shrubs were sampled, as listed in Text S1). All samples were stored frozen.

Before extracting the DNA, the samples were preprocessed. For honey bees, to extract DNA of microbes from on and in the body of honey bees, homogenized honey bee tissue was used. For honey, 10 g of honey was diluted to 30 ml of DNA clean water, the sample was centrifuged, and the supernatant was discarded. For flowers, a water-bath sonicator and vortexing were used to detach the microbes. After removing most of the flower tissue, the sample was centrifuged and the supernatant discarded (Text S1).

The DNA was extracted in the same way from all samples with the DNeasy Plant Mini Kit (Qiagen, Germany). To identify bacteria, a part of the gene region 16S was amplified and for fungi a part of ITS2, with taxon-specific primers (16S_515FB and 16S_806RB [45, 46] and ITS2-F and ITS2-R [47], respectively). To identify plants in honey samples, a part of ITS2 was amplified with plant-specific primers ITS2-F and ITS2-R [48, 49]. After the initial amplification with tagged primers, the samples we labeled with unique dual‐index combinations in a second PCR and pooled libraries of the three gene regions were formed. The libraries were sequenced with Illumina MiSeq V3 (Text S1). The bioinformatics processing of reads followed Kaunisto et al. [50] (see details of the bioinformatic processing in Text S1). For the bioinformatic processing, the reads of all samples were combined per gene region. The reads were truncated, merged, and quality controlled. Primers were removed, and the reads were dereplicated and singletons were removed. The reads were denoised to ZOTUs [42]. The taxonomic assignation of ZOTUs was done by comparison against a specific reference database for each gene region (Text S1).

### Statistical Analyses

#### Comparison of Bacterial and Fungal Communities in Honey Bee, Honey, and Flower Samples

Statistical analyses were performed in R v.4.3.1 [51]. All the analyses were run separately for both bacterial and fungal communities, at the taxonomic level of ZOTUs. Additionally, the main analyses were run with families to assess the robustness of patterns in regard to the taxonomic level used. All the analyses were based on presence–absence data, unless separately indicated that relative read abundances were used. Thus, this does put emphasis on rare taxa even though the rarest ones were omitted from the analyses (see below).

To answer how much of the bacterial and fungal taxa are shared among the honey bee, honey, and flower samples, in comparison to being found uniquely in each, we drew Euler diagrams with the *eulerr* package [52]. To determine the difference in the bacterial and fungal community composition between honey bee, honey, and flower samples, we applied the multivariate homogeneity of group dispersion of Bray–Curtis dissimilarity using “*betadisper*” in package *vegan* 2.6–4 [53]. A significant difference in the multivariate distance between the plots and the sample type-specific centroid indicates heterogeneity in variance in species composition among communities, i.e., difference in beta-diversity. For this and the following analyses assessing factors affecting the community compositions, we omitted samples with less than 2000 reads of the targeted taxonomic group (bacteria or fungi), to avoid including samples with few sequences due to amplification or sequencing issues. Taxa occurring in less than three samples were omitted from the community analyses.

To explore the factors influencing bacterial and fungal communities, we built three separate models: (1) to test the spatial and temporal effects on the microbial communities of honey bees, honey, and flowers; (2) to test the spatial and temporal effects and flower identity on flower microbial communities; and (3) to test the effects of the most-visited flowering plants present in honey samples on microbial communities of honey bees and honey (for details, see Text S2). The effects of different variables on community composition were estimated with redundancy analyses (RDAs) using “*rda*” in *vegan* 2.6–4 [53]. Last, to examine how the bacterial and fungal communities in honey and flower samples explain the respective communities in honey bees and potential transmission pathways, we constructed structural equation models (SEMs). We modeled direct pathways from flowers to honey, and from both honey and flowers to honey bees, and an indirect path from flowers to honey bees through honey. We built separate models for bacterial and fungal communities. For the modeling, we used the proportions of each sample type (honey bee, honey, flower) where a certain ZOTU was present (567 and 1072 ZOTUs for the bacterial and fungi, respectively). The models were fitted using package *lavaan* [54].

## Results

We collected 98 honey bees, 99 honey, and 143 flower samples in 2021 (Text S3 and Table S1). After the bioinformatic processing, 98 and 97 honey bee samples had filtered bacterial and fungal reads, respectively. All honey samples had bacterial, fungal, and plant reads. Of the flower samples 110 and 133 had bacterial and fungal reads, respectively, due to majority of the flower samples’ reads being of plant origin instead of the targeted bacteria or fungi (only 8.1% and 40.3% of the filtered reads, respectively; Tables S2 and S3). Of all the filtered reads assigned to bacteria, fungi and plants, 99.2%, 70.8%, and 100.0%, respectively, were assigned to a family (Table S2).

### Bacterial and Fungal Communities’ Composition of Honey Bee, Honey, and Flower Samples

The frequency of occurrence of bacterial and fungal families and genera among samples varied largely between the different sample types (Tables S4 and S5). As an example, the bacterial genera, which the five core honey bee gut microbial species clusters belong to (*Lactobacillus* Firm-4 and Firm-5, *Snodgrassella alvi*, *Gilliamella apicola*, and *Bifidobacterium* spp. [55]), were found in all honey bee samples, as expected. They were found in a far smaller proportion of honey samples and were nearly absent in the flower samples.

**Fig. 1:**
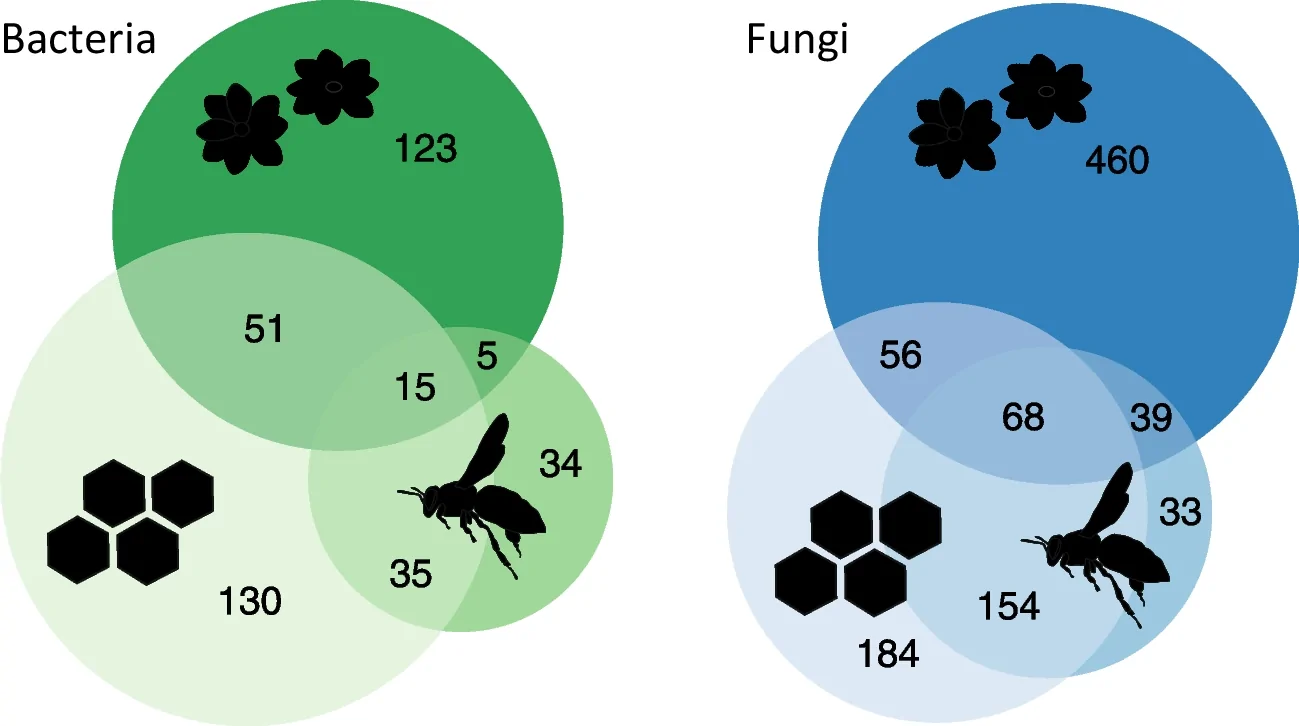
Euler diagrams showing the number of shared and non-shared bacterial and fungal ZOTUs between honey bee, honey and flower samples, shown with the symbols, with all samples and all taxa included

The proportions of shared ZOTUs and families between honey bee, honey, and flower samples varied between bacteria and fungi (Figs. 1 and S1). The portion of taxa shared between honey bee and honey samples for bacteria is clearly smaller than that of fungi (56.2% and 75.5% of honey bee ZOTUs detected in honey samples, respectively). Vice versa, there were more bacterial than fungal sharing between flowers and honey (34% and 19.9% of flower ZOTUs detected in honey, respectively). All these patterns are consistent also at the family level (Fig. S1). Meanwhile, honey bees shared less bacteria than fungi with flowers (22.5% and 36.1% of honey bee ZOTUs detected in flowers, respectively). Only 3.8% of bacterial ZOTUs and 6.8% of fungal ZOTUs were detected in all three sample types.

**Fig. 2:**
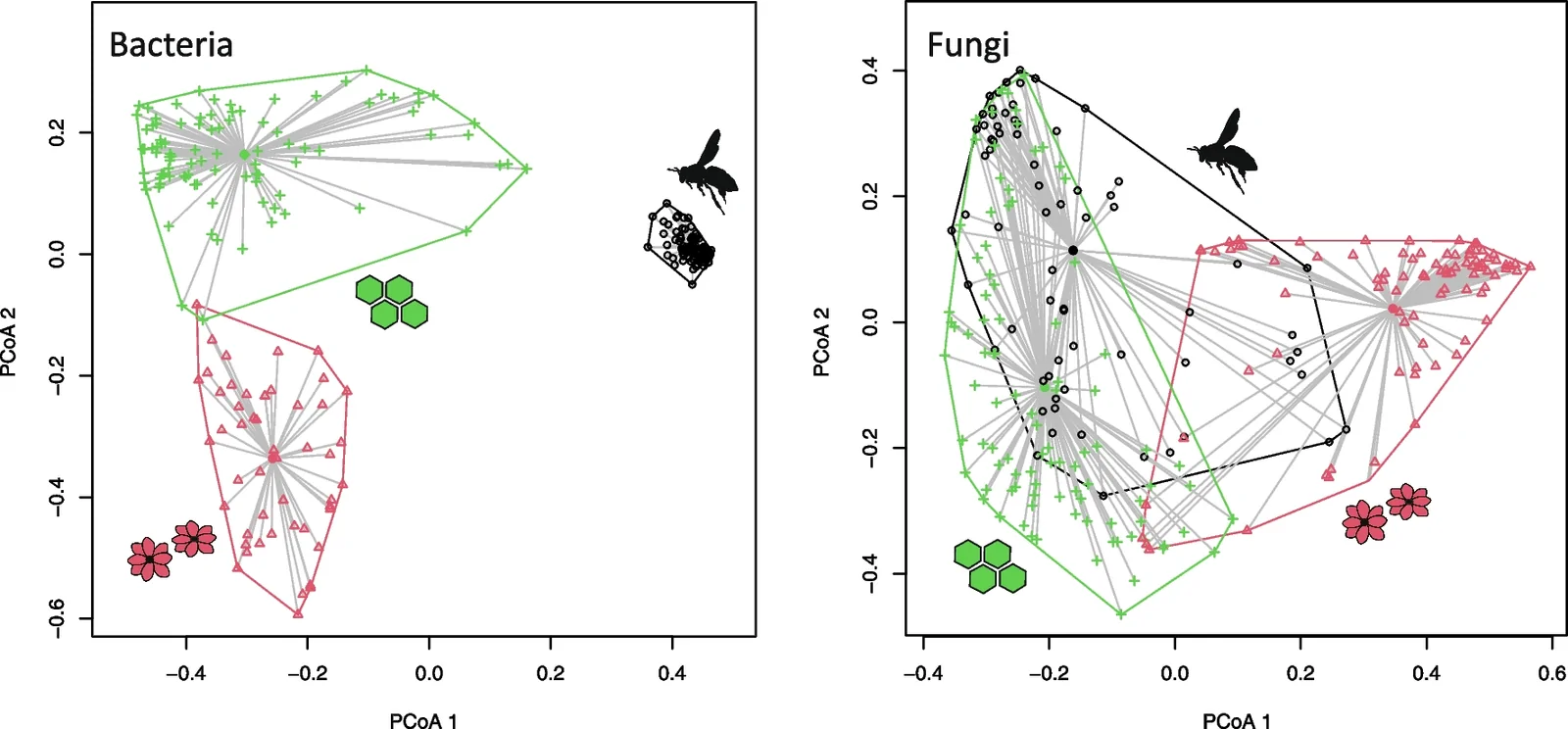
Similarity of bacterial and fungal communities in honey bee, honey, and flower samples based on principal coordinates analyses, with presence-absence data, for the ZOTUs. Samples with less than 2000 reads and taxa with less than three occurrences are omitted from the analyses. The first and second PCoA axes explained 33.3 and 9.6% and 21.9 and 11.3% of the variation of the bacterial and fungal communities, respectively (Table S6)

While assessing the similarity of bacterial and fungal community composition between sample types, bacterial communities are clearly more clustered than the fungal communities, for which the compositional overlap is stronger (Fig. 2). The group dispersion (β-diversity) of the bacterial communities of honey bee samples is lower than that of honey or flower samples, with the average distance to centroids being 0.17, 0.59, and 0.47, respectively, while it is of similar magnitude for fungal communities, ranging between 0.50 and 0.57 (Table S6, Fig. 2). The community composition of different sample types is more similar when considering families (Fig. S2). Yet, the overall patterns remain despite the taxonomic level as well as when considering also the relative read abundances, not only presence-absence data (Fig. S2).

### Different Spatiotemporal Structure of the Microbiota of Honey Bees, Honey, and Flowers

All the models exploring the factors influencing bacterial and fungal communities (Text S2) were statistically supported, while the adjusted *R*^2^ values varied from 2.8% for bacterial communities of flowers to 18.3% for fungal communities of honey bees (Table S7). Based on the variance partitioning, the variance accounted for by the spatiotemporal variables, by region and month together, vary largely among the bacterial and fungal communities, ranging from 2.8% (month: 1.0% + region: 1.8%) for the bacterial communities of flowers to 18.6% (month: 13.8% + region: 4.8%) for the fungal communities of honey bees (Table 1). The methodological bias quantified by the number of reads accounted for a minor proportion of the total variation, ranging from 0.5 to 2.6% (Table 1).

**Table 1:** Results of the redundancy analyses for the microbial communities of the different sample types, considering the communities of ZOTUs and using presence-absence data. Below are shown the results of the variance partitioning between the fractions. Detailed results of the model and the ANOVA partitioning are given in Table S7

	Bacteria		Fungi	
Fractions	Df	adjR2	Df	adjR^2^
Honey bees				
Month	2	7.6%	2	13.8%
Region	2	1.8%	2	4.8%
Reads	1	2.6%	1	0.5%
Residuals		90.0%		81.1%
Honey				
Month	2	8.8%	2	6.9%
Region	2	9.0%	2	9.3%
Reads	1	3.3%	1	2.3%
Residuals		81.7%		81.5%
Flowers				
Month	2	1.0%	2	8.1%
Region	2	1.8%	2	0.6%
Reads	1	1.9%	1	2.5%
Residuals		95.3%		89.4%

Of all the sample types and communities tested, only the bacterial communities in flowers were not affected by the time of the summer (Table S7). The two types of microbial communities in the honey bee samples are not structured in the same way by the spatiotemporal variables, with a greater effect of the time of the summer and the region on fungal than on bacterial communities (Tables 1 and S8). The effect of time is either of the same order of magnitude or much greater than the effect of space (Table 1). The results based on the ZOTUs are supported also by models run at the family level (Table S8).

Including the identity of the focal plant, as the plant family it belongs to, in the model for flower samples (Text S3), increased the proportion of the variance explained (Table S9). In the model, the contribution of the plant family is clearly higher than the one of spatiotemporal variables, yet for bacteria, the plant family was not statistically significant for the ANOVA partitioning (*p* = 0.066; Table S9). The impact of the flower families used by the honey bees is smaller on the honey bees’ bacterial community but of similar magnitude for that of honey and the fungal community in both honey bee and honey samples (Text S3, Table S10).

### Bacteria and Fungi have Different Dispersal Pathways between the Microbiota

**Fig. 3:**
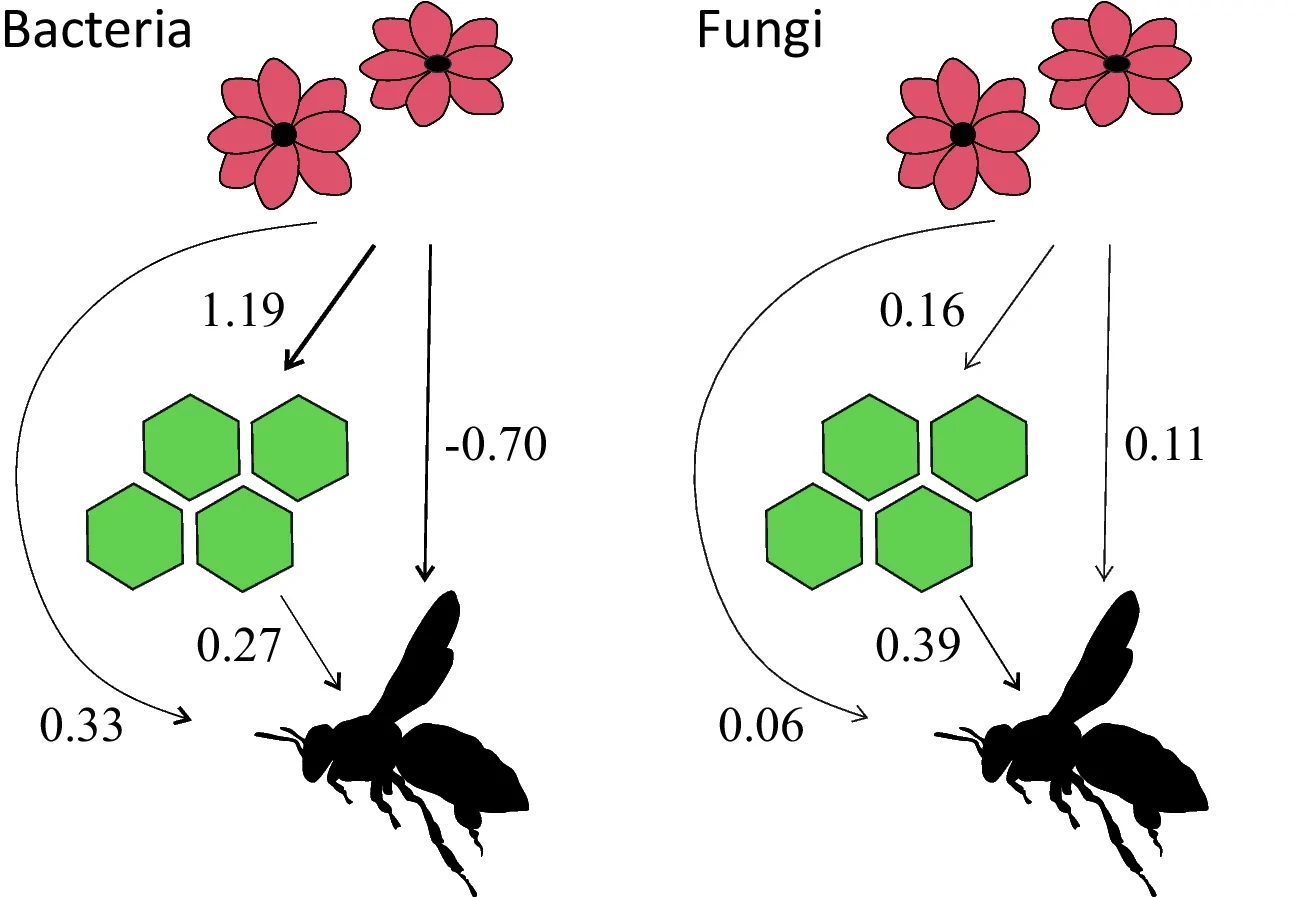
Pathways of the bacterial and fungal ZOTUs from flowers to honey and to honey bees based on the SEM analyses. The direct lines show the direct pathways, while the circle shows the interaction of the pathways flower-to-honey and honey-to-honey bees. All the pathways shown are statistically supported

Bacterial communities transfer from flowers to hive, represented by honey, more than fungal ones (Fig. 3, Table S11). The direct pathway from hive to honey bees is of similar importance for both bacteria and fungi, but the indirect pathway from flowers to honey bees through hive is much stronger for bacteria than for fungi. Meanwhile, bacterial taxa are not transferred directly from flowers to honey bees, while for fungi, this also happens. These patterns prevail also when analyzing the data at the taxonomic level of families and, in addition, the pathways are stronger except for the direct pathway from hive to honey bees in fungi (Fig. S3).

## Discussion

We found the bacterial and fungal microbiota of honey bees, their honey, and the surrounding flowers to be partially shared even at the level of individual DNA-strains, indicating microbial dispersal between them. However, the dispersal routes differed between fungi and bacteria. While most bacterial ZOTUs in or on the honey bees are not shared with or obtained from their environment, nearly one fourth of them are, as we observed, 22.5% of the honey bees’ bacterial ZOTUs also in the flower samples.

Considering the bacteria found in honey samples, representing microbes living in the hive and brought into the hive by the honey bees from their environment, only few of the bacterial taxa were also found in and on honey bees. This is in line with previous research showing that gut bacteria contribute the vast majority of microbial DNA sequences of honey bees, while only a little microbial DNA originates from the honey bees outside surfaces in a DNA metabarcoding-based assessment when comparing samples of whole honey bees, bee guts, and bee individuals from which the guts were removed [44]. The highly specialized bacterial community of honey bee guts [56, 57] is known to be mainly obtained through the interactions with colony members, but also from hive materials [57]. The gut as an environment for microbes is very different from hive surfaces, highlighting how exposure to microbes is only one step of the microbiota formation [58]. In contrast to the gut bacteria, the bacteria on the honey bees could be expected to be more shared with their hive, their food sources, and other parts of their environment, and thus, the bacteria found shared among honey bees, honey, and flowers in our study, as an example of ZOTUs from the genera *Pseudomonas* (*Pseudomonaceae*), and *Spiroplasma* (*Spiroplasmataceae*), are likely to be mainly from the surfaces of the bees. Members of both these genera have been associated both with honey bees and flowers and with members of *Pseudomonas* likely to prevent diseases while members of *Spiroplasma* are likely to cause such [25, 59, 60].

Interestingly, the fungal communities of the honey bees and their honey show a totally different pattern from that of bacteria, as the majority of the taxa are shared, and the communities are highly similar. As flowers provide the main source of food for bees, the fungi found in nectar and pollen can directly contribute to microbiota in bee nests and guts, and thus, the likely source for them has been proposed to be the plants [34]. On top of being exposed to fungi arbitrarily while foraging, honey bees have been observed to collect fungal spores from plant surfaces, to complement their nutrition with these [34, 61]. Fungi in honey bees’ food stores also changes in time, initially resembling those of flowers but decreasing in diversity and abundance over time. This has been found to happen in fungi associated with stored pollen, i.e., bee bread [62, 63]. We here examined DNA traces in freshly covered honey, presenting a sample of any DNA present in the hive as the honey would have been prepared from nectar spread to a large number of open combs, allowing any DNA traces to enter and be stored in it. Yet, sampling bee bread and hive surfaces directly for microbes would possibly complement the list of microbes encountered in and brought to the hive and lead to discovering a greater overlap between microbial communities [64] than we discovered by sampling only honey.

Overall, to our knowledge, other studies have not examined the difference in sharing of fungi and bacteria of honey bees with their honey and environment. Most research in regard to microbes of bees has focused on bacteria, yet fungi may be pathogenic, living in food storages causing spoilage, as well as commensal or even mutualistic [34]. Especially, the non-pathogenic fungal members of honey bee gut microbiota are mainly unstudied [24]. Yet, ecologically interesting fungal taxa are common members of the fungal communities of both honey and honey bees also in our study, for example, the fungal honey bee pathogens, belonging to the genera *Ascosphaera* and *Aspergillus* [27, 65], and yeasts like *Metschnikowia* living in nectar and moist honey [66]. Most microbiota studies typically focus on a single host type [23] and thus cannot explore the relationships between the different microbiota. Here, through studying the microbiota within a connected system, we were able to address the multitrophic dispersal pathways of the microbes. While many of the fungal or bacterial taxa observed here in or on bees, honey, and flowers may not have an ecologically meaningful role on the specific substrate or host on which they were observed, their presence or absence tells us about the relationships of bees, honey, and flowers and where different microbes may be encountered. Our examination of the patterns based on presences and absences does delimit the applicability of our results as the abundances of fungi and bacteria are likely to vary greatly in different parts of the pollination system, with as an example bacteria being highly more abundant in honey bee guts than fungi [3, 24]. Yet, with the occurrences of taxa, most of the bacterial and fungal taxa of the flower microbiota were not detected as members of the microbiota of either honey or honey bees, and both the bacterial and fungal community compositions of flowers were totally distinct from the communities of honey bees and only overlapping a little with the communities of the honey. These results contradict recent findings proposing that the dispersal of microbes between plants, mediated by pollinators, is an important factor shaping microbial communities of plants and the pollinators [23]. This could be due to a lesser role of dispersal per se, in comparison to the characteristics of the habitat and the interspecific interactions with the microbes already inhabiting the habitat, in the formation of a microbiota, in other words, the filtering of microbial taxa [23].

The identity of the flower visitor plays a central role in this dispersal [10], yet based on our results, *Apis mellifera* does not play a substantial role in shaping the bacterial or fungal communities of flowers, although they might have a large impact on the transfer of certain microbes. In support of this minor role, Ushio et al. [67] found that the impact of a visit of a pollinator is small on the diversity of bacteria on flower surfaces, although different insects do leave differing microbial footprints. Yet, for the honey bee, it has been shown their visits increase the diversity of bacteria on grapefruit, *Citrus paradisi*, flowers [21] as well as the diversity of both bacteria and fungi on *Salix* inflorescences [68]. As microbes may change nectar properties, as an example by changing the sugar composition and decreasing the sugar concentration [69], a change in a flower’s microbiota might impact pollinator visits, and this may change the sharing of microbes among the flowers and pollinators [7]; thus, even small contributions to the microbiota of flowers by the honey bee may be impactful.

### Spatiotemporal Structure of Microbiota

We found that both the sampling region and time of the season influenced the studied bacterial and fungal communities. Still, the bacterial communities of honey bees of all the sampled colonies in the different regions, and in different months, were very similar. The same has been found in another study, where the bacterial communities of whole honey bee individuals varied only slightly over the course of a honey production season and not at all between different hive types [70]. This is consistent with the honey bee microbiota being strongly dominated with the restricted set of gut bacteria [56, 71], yet the bacteria on the body surfaces could be more exposed to temporal changes.

Our results show that the relative abundance of the honey bees’ most abundantly used plant families affect both the bacterial and fungal communities of the honey bees and the honey. This is in line with previous studies showing varying diversity in microbial composition across different flower species and flower characteristics, such as volatile organic compounds, defining which microbes inhabit them [72, 73]. Sampling time and region largely define the community of flowers available to honey bees to select from, but there, the relative proportions of different plant families honey bees have used, have a strong impact on the microbiota of honey and honey bees.

### Honey Bees Acquire Bacteria and Fungi Differently from Honey and Flowers

When considering the pathways of microbial taxa from the surrounding microbe pool of flowers to the honey bees and the honey, fungal taxa are introduced into the honey bee microbiota directly from flowers and the hive, and from the flowers through the hive. For bacteria, taxa are transferred from flowers to honey, but not directly to honey bees at all.

Honey bees spend the first weeks of their lives in the hives and have very similar bacterial communities with each other, with progressive changes as they age [74, 75]. Our study further suggests that only a minority of the bacteria occurring in the hive or in the surrounding environment of hives becomes a part of the honey bee microbiota, reinforcing the hypothesis of an ecological filter through selection due to biotic interactions [6].

Meanwhile, the larger variability in their fungal microbiomes shows that there is more room for environmental acquisition, and honey bees would acquire fungal taxa both from the honey and the flowers based on our analyses. Here, the role that bacteria versus fungi play in honey bees is likely to be of importance, as fungi are only rarely part of the gut microbiota of honey bees [76]. Thus, fungi may inhabit other structures of honey bees and especially the surfaces, allowing for a proportionally larger sharing of them. We found that flowers, hive, and flowers through hive can act as dispersal routes of microbes to honey bees. This supports the view of hive as an extended organism to honey bees in terms of their microbiota [19] and offers greater understanding how the different communities of microbiota are shared and interact, yet, taking into account all the above, more research on especially the sharing and transfer of fungi by honey bees is needed, to understand the formation and functioning of the full microbiota of this system.

## Conclusions

Honey bees’ movements between flowers and the hive make the hives potential hotspots from where the microbes might be dispersed further directly (e.g., to flowers) and indirectly (e.g., to other pollinators through flowers) by the honey bees. While several studies have shown microbe sharing to take place between flowers and pollinators, based on our results, the encounters of honey bees and flowers shape the others’ microbiota relatively little, this being especially revealed when looking at the level of individual strains of microbes, as here with ZOTUs. While the microbiota of the honey bee, the hive (as represented by DNA of microbes in honey), and the surrounding flowers are all impacted by the time and region in a relatively similar magnitude, it appears that their bacterial and fungal communities are fairly distinct. Considering how different honey bees, honey, and flowers are as habitats for bacteria and fungi to live in, in regard to nutrient composition, environmental conditions, and longevity, this result is not contradictory despite the sharing of microbes [77]. While the gut microbiota of honey bee is known to be specialized, so are those of many other bee species, especially social ones, which have a route to share the microbes to offspring, and thus, provide the microbiota an opportunity to evolve within the host species [5, 57]. Consequently, our results of horizontal microbe sharing in the world’s most dominant pollinator are likely to some extent be generalizable to other closely related social bees. However, many other pollinator groups acquire their gut microbiota more opportunistically, leaving the role of different bees, flies, butterflies, and moths in microbe sharing of pollination networks to be further examined.

## Supplementary Information

Below is the link to the electronic supplementary material.

Supplementary file1 (DOCX 847 KB)

## Data Availability

The sequence datasets generated during the current study are available in the Sequence Read Archive repository, in the BioProject PRJNA930592 (https://www.ncbi.nlm.nih.gov/sra/ PRJNA930592).
