# Supplementary material for: Distinct Communities and Differing Dispersal Routes in Bacteria and Fungi of Honey Bees, Honey, and Flowers
Source: Microb Ecol. 2024 Jul 30;87(1):100. doi: 10.1007/s00248-024-02413-z (PMC11289361; doi:10.1007/s00248-024-02413-z)
Supplement: Supplementary file 1 — Supplementary file1 (DOCX 847 KB) [file 248_2024_2413_MOESM1_ESM.docx]

**Distinct communities and differing dispersal routes in bacteria and fungi of honey bees, honey and flowers**

**Supplemental material**

Mikko Tiusanen^1,2^, Antoine Becker-Scarpitta^1,3^, Helena Wirta^1,4*^

^1^University of Helsinki, Department of Agricultural Sciences, Helsinki, Finland

^2^Eidgenössische Technische Hochschule Zürich (ETH Zürich), Department of Environmental Systems Science, Zürich, Switzerland

^3^CIRAD, UMR PVBMT, Saint Pierre, La Réunion, France

^4^Umeå University, Department of Ecology and Environmental Science, Umeå, Sweden

^*^corresponding author: [helena.wirta@helsinki.fi](mailto:helena.wirta@helsinki.fi)

**Summary**

**Texts**

- Text S1. *Sampling, sample preprocessing, DNA extraction, target amplification, sequencing and bioinformatics processing*
- Text S2. *Quantification of factors affecting composition of microbiota*
- Text S3. *Bee, honey and flower samples*

**Figures**

- Figure S1. Euler diagrams showing the number of shared bacterial and fungal ZOTUs and families between bee, honey and flower samples, shown with the symbols, with all samples and all taxa included. Bacterial and fungal ZOTUs and families are shown.
- Figure S2. Similarity of bacterial and fungal communities in honey, bee and flower samples based on principal coordinates analyses, showing the first two axis, for the taxonomic level of ZOTUs and families, for both presence-absence and relative read abundance data. Samples with less than 2000 reads and taxa with less than three occurrences are omitted from the analyses. The panels with ZOTU and presence-absence data are identical to the ones shown in Fig. 2.
- Figure S3. Pathways of the bacterial and fungal ZOTUs and families from flowers to honey and to bees. The panels with ZOTUs are identical to the ones shown in Fig. 3.

**Tables**

- Table S1. List of flowering plants sampled, with the total number of samples per species as well the the number of samples with data for bacteria (16S gene region) and fungi (ITS gene region).
- Table S2. Number of reads after different steps of the bioinformatics processing for the three gene regions, with the number of reads assigned to different taxonomic levels.
- Table S3. Number of samples with data for bacteria, fungi and plants, for the three different sample types bees, honey and flowers. The average number of reads per sample and the standard deviation is given, based on all the samples with sequence reads for the concerned gene region.
- Table S4. The frequency of occurrence (FOO) of bacterial families and genera detected in the different sample types. The number of occurrences across the whole data set for each taxon is also shown.
- Table S5. The frequency of occurrence (FOO) of fungal families and genera detected in the different sample types. The number of occurrences across the whole data set for each taxon is also shown.
- Table S6. Results of the multivariate homogeneity of group dispersion analyses and the principal component analyses, for both the presence-absence (PA) and relative read abundance data (Abundance), as shown in Figs 2 and S2.
- Table S7. Results of the redundancy analyses for microbial communities of the different sample types, for the communities of ZOTUs based on presence-absence data, as well as the ANOVA results for the variance partitioned.
- Table S8. Results of the redundancy analyses for microbial communities of the different sample types, for the communities of families based on presence-absence data, as well as the ANOVA results for the variance partitioned.
- Table S9. Results of the redundancy analyses for the flower samples, considering the family of the flower sample in the model as well, for both microbial communities based on ZOTUs with presence-absence data, as well as the ANOVA results for the variance partitioned.
- Table S10. Results of the redundancy analyses for bees and honey samples, considering the relative proportions of eight most abundantly used flower families based on the honey samples, for each hive at each time point. The results of the redundancy analyses are shown for bacterial and fungal communities based on ZOTUs, with presence-absence data, as well as the ANOVA results for the variance partitioned.
- Table S11. Detailed results of the SEM pathway analyses for bacterial and fungal ZOTUs and families from flowers and honey to bees.

Text S1. *Sampling, sample preprocessing, DNA extraction, target amplification, sequencing and bioinformatics processing*

*Sampling*

To study the microbiota of honey bees, honey and flowers, we studied 36 honey bee colonies (29 colonies at the end of the summer) and the surrounding flowering plants in South-Finland. The hives were located in six apiaries, with two to eight hives per apiary. The six apiaries formed three pairs where the apiaries were less than 2 km apart and the apiary pairs (called hereafter region) were 10-15 km apart from each other [as in 1]. As honey bees only rarely forage more than 3 km away from their hive, we consider the floral resources to be shared within a region, while between regions not.

To examine the temporal dynamics of the composition of the microbiota, we collected bee, honey (representing the hive) and flower samples in June (8.-13.6.), July (9.-14.7.) and August (10.-13.8.) of 2021. For the microbiota of honey bees, we collected one deciliter of a mixture of nurse and forager bees into a plastic bag from each hive and froze them immediately.

Of these, a subsample of six individual bees were homogenized to determine microbiota in and on honey bees. We used whole individuals, as the whole-body microbiota represents the gut microbiome and also captures the whole-body microbial exposure [2]. For the honey microbiota, we collected a spoonful of newly covered honey from three frames from each hive, to cover the variation in honey within a hive at a time. For microbiota of flowers, we sampled abundantly flowering herbaceous plant and shrub species (two to ten species) in each different habitat type in each region. Six or seven habitat types had been identified and assessed in the vicinity of the apiaries per region [1]. We also sampled occasional rare species known to attract honey bees as well as cultivated species in the vicinity of the apiaries. Depending on the size of the flowers, nine to twelve individual flowers or inflorescences were collected directly into 99% EtOH. All samples were stored frozen.

*Preprocessing*

Before extracting the DNA, the samples were preprocessed. For bees, to extract DNA of microbes from on and in the body of honey bees, we used a sample of homogenized honey bee tissue. For each bee sample, two subsamples of three bee individuals were placed with DNA-clean forceps into a 2 ml tube with one metal bead of 3 mm Ø and one of 4 mm Ø. The tubes were frozen in liquid nitrogen for 5 minutes, after which they tissue was homogenized for 2 x 30 s with 30 Hz (Mixer Mill MM 400, Retsch, Germany). The samples were let to melt for 5 min in room temperature and they were further homogenized with 2 x 30 s with 30 Hz. From each of two subsamples about 50 mg of the homogenized tissue were taken into a new tube, forming the sample for DNA extraction. For honey, the honey sample collected from three frames was mixed and 10 g of honey was diluted to 30 ml of DNA clean water (MilliQ, Merck KGaA, Germany a) in a 50 ml tube. The honey was let to dissolve into the water for 30 min in 60 °C. The samples were the centrifuged at 8000 G for 60 min (Centrifuge 5810 R, Eppendorf, Germany), after which most of the supernatant was discarded and the pellet was transferred to a 2 ml tube. The 2 ml tube was further centrifuged at 11 000 G for 5 min and the remaining supernatant was removed. For flowers we used a water-bath sonicator and vortexing to detach microbes from the flowers. First, the tubes were placed in a water-bath sonicator (Branson 2510E-MTH, Branson Ultrasonics Corporation, USA) for five minutes. Then, a metal bead of 3 mm Ø was added and the sample was vortexed for 5 minutes at level 5 (Vortex-Genie2, Scientific Industries Inc., USA). After this, large flower tissue pieces and the bead were removed with DNA-clean forceps. The 50 ml tubes were centrifuged at 8000 G for 60 min (Centrifuge 5810 R, Eppendorf, Germany), after which most of the supernatant was removed and the pellet was transferred to a 2 ml tube. The 2 ml tubes were centrifuged with 14 000 G for 15 min, after which the supernatant was removed. To dry out the remaining ethanol before DNA extraction, the tubes were placed in oven for 30 min at 60 °C with the lids open, covered by tissue. All the preprocessed samples were stored in freezer until DNA extraction.

*DNA extraction, amplifications and sequencing*

To minimize differences in the processing of samples, total DNA was extracted in the same way from all samples with the DNeasy Plant Mini Kit (Qiagen, Germany). The protocol of the kit was modified as follows. First, the pellet was resuspended in 400µl of buffer AP1, and then 4µl Rnase, 4µl proteinase K (20mg/ml, Macherey-Nagel) and one 3 mm tungsten carbide bead was added to each sample tube. The sample was then disrupted 2 x 2 min 30 Hz (Mixer Mill MM 400, Retsch, Germany). DNA extraction then followed the protocol with the exception of skipping the QIAshredder column step to avoid loss of DNA. With the extraction of each sample type, 2-3 DNA extract controls were included. In the laboratory all the steps before the amplifications were done in a laminar hood wiped with ethanol and cleaned of DNA with 1 hour UV light every night. We only used DNA-free tubes, pipet tips and PCR plates as well as DNA-free water.

The initial amplifications were done with a total volume of 10 μl, each containing 5 μl MyTaq Red Mix (Bioline, London, UK), 1.3 μl DNA-free water, 0.3 μl of each primer (10 μM) and 3 μl of DNA extract. PCR cycling conditions were as follows, with primer-specific annealing temperatures. For bacteria with tagged primers 16S_515FB and 16S_806RB [3, 4] annealing was at 50 °C, for fungi with ITS2-F and ITS2-R [5], annealing was at 47 °C, and for plants with ITS2-F and ITS2-R [6, 7] at 55 °C. The initial denaturation was for 3 min at 95 °C, followed by 28 cycles of 30 s 95 °C (denaturation), 30 s 47-55 °C (annealing), 30 s 72 °C (extension), and ending with final extension for 7 min at 72 °C. For amplifying fungi from bee samples, we used 35 cycles as with 28 cycles most samples showed no clear amplification based on assessment on the band on agarose gel. To minimize initial bias of amplification, each reaction was carried out as two replicates. All the amplicons were checked on a 1% agarose gel and imaged with a BioRad imager to check the reaction had worked and the DNA and PCR controls were clean. The PCR replicates were combined before library-PCR as 1.3 μl of each PCR product replicate. Illumina‐specific adapters and unique dual‐index combinations for each sample was used [8]. The library PCR had a total volume of 10 μl, each containing 5 μl MyTaq Red Mix (Bioline, London, UK), 0.3 μl of reverse primer (10 μM), 2.1 μl of forward primer (1.43 μM) and 2.6 μl of the locus-specific combined 1^st^ PCR product. PCR cycling conditions were as follows, the same for all gene regions for the library PCR. Starting with 4 min 95 °C to denature, followed by 15 cycles of 20 s 98 °C, 15 s 60 °C and 30 s 72 °C, and ending with 3 min 72 °C. DNA libraries were pooled per gene region and per 96 samples, and concentrated using a SPRI bead protocol. The concentrated pooled sample was loaded on 1% agarose gel (Agarose tablets + TAE) and run with 90 V for 120 minutes. The target bands were cut on UV light and the pooled sample was cleaned from gel with the PCR and Gel CleanUp Kit (Macherey-Nagel), diluted in 2 x 20 μl of the elution buffer provided in the kit. The DNA concentration of the cleaned pools were measured with Qubit 2.0 (dsHS DNA Kit, ThermoFisher Scientific).

Based on the compatible lengths of the targeted gene regions, the pools of 96 samples were combined in equimolar ratios and sequenced in three MiSeq sequencing runs with v3 chemistry with 600 cycles and 2 x 300 bp paired-end read length.

*Bioinformatics*

The bioinformatics processing of reads followed Kaunisto et al. 2020 [9]. For the processing the reads of all samples for this study were combined per gene region. The processing of reads was started by truncating the reads to 220 bp for 16S and 240 bp for ITS2 for both plant and fungi. This was done to cut off lower quality ends before merging the paired ends for each gene region using VSEARCH [10] with a maximum of 80 differences allowed for overlap and a minimum assembly length of 150 bp. The merged reads were quality controlled by fastq_maxee, with maxee = 3. The merged and quality controlled reads were only retained if they contained the expected primers at each end. Primers were removed using cutadapt with a maximum of 0.2 error rate for primers, and reads were kept with minimum length of 100 bp after primer removal. The reads were dereplicated and singletons were removed. The reads were denoised to zero-radius operational taxonomic units (ZOTU) using with unoise3 with USEARCH [10]. A ZOTU table was build and the taxonomic assignation of ZOTUs was done by comparison against a specific reference database for each gene region with VSEARCH. 16S for bacteria were compared against the 16S RDP reference database, version 18 [11], ITS for fungi against the UNITE fungal ITS reference database, version 10.05.2021 [12] and ITS2 for plants against an ITS2 reference database from PLANTiTS, accessed 21.3.2022 [13].

To remove possible misassigned reads and false positives, due to contamination, we further filtered the reads in ZOTUs [following e.g. , 14, 15]. As small numbers of reads were found in all controls, reads were removed if they were less than the maximum number of reads from the DNA extraction or PCR negative controls from all the samples for each ZOTU. ZOTUs with less than 0.05% of the total read number of that sample were removed, as well as ZOTUs with less than 10 reads were removed.

Text S2. *Quantification of factors affecting composition of microbiota*

To quantify the factors affecting the bacterial and fungal community composition, we used different partial canonical analyses (redundancy analyses, RDA) with the function “*rda*” of the package *vegan* 2.6-4 [16]. First, we tested separately for each sample type (honey bee, honey, flowers) the spatio-temporal structure of each community *i* with the following model:

equation 1 RDA*_sample_type_ (*community*_i_* ~ month + region + Condition(reads))

The explanatory factors include temporal variation (month), spatial influence among different sites (region) and a term to take account for methodology resulting in different numbers of sequence reads obtained per sample (reads).

Second, to assess how plant identity impacts the bacterial and fungal community composition of flowers, we run an alternative model for the flower samples only, including the identity of the flower at the family level (plant_family) :

equation 2 RDA*_flowers_* (community*_i_* ~ month + region + plant_family + Condition(reads))

Third, considering the relative read abundances of the eight most frequently and abundantly occurring plant families across the honey samples, we build another model quantifying how different plant choices of the honey bees impact the bacterial and fungal community composition *i* in bee and honey samples only. As the occurrence and abundance of plants are strongly influenced by the time and location, we assess the effects of different plant families separately, with the following model:

equation 3 RDA*_sample_type_*(community*_i_* ~ Brassicaceae + Rosaceae + Fabaceae + Salicaceae + Boraginaceae + Apiaceae + Asteraceae + Ericaceae + Condition(reads))

The variation associated with each fraction of the canonical models were quantified by variation partitioning the function “*varpart*” and adjusted R^2^ were calculated with the function “*RsquareAdj’* from the package *vegan* [16].

The usage of Brassicaceae, Fabaceae and Salicaceae have a significant impact on both the bacterial and fungal communities in both honey bee and honey samples. On top, the usage of Apiaceae shows as an important plant family affecting bacterial and fungal community composition found in honey samples. Similarly, Asteraceae is important for the fungal community composition in honey bee samples (Table S11). Yet, the strength of the impact of the different plant families’ usage varies for bacterial and fungal communities (Table S11).

Text S3. *Bee, honey and flower samples*

At the beginning of the sampling there were 36 hives from which we sampled, but unfortunately nearly one third of the colonies were left without a queen (queen had died or swarmed) before the second sampling, so 29 hives remained for sampling for the rest of the season 2021. In total we collected 99 honey and 98 bee samples for 2021.

For flowers close to the apiary regions, we collected in total 143 flower samples, 42, 61 and 40 samples in June, July and August, respectively, in 2021. A sample of three cultivated plants found flowering in the vicinity of the hives were *Linum usitatissium*, *Brassica* sp. and *Solanum tuberosum*, were collected in July and are included (Table S1).

After the bioinformatics processing, 307 samples had data for the bacterial 16S region, 329 for the fungal ITS region and 99 samples for the plant ITS2 (Table S2). Of the 143 flower samples, only 110 and 133 samples had data for the bacterial 16S and fungal ITS, respectively. This was due to the high fraction of the flower samples’ reads originating from plant DNA, instead of the targeted bacteria or fungi (only 8.1% and 40.3% of the filtered reads, respectively). Instead, all the 99 honey samples had bacterial, fungal and plant data, and all the 98 bee samples had data for bacterial 16S, and all but one bee sample for fungal ITS.

Of all filtered 16S reads assigned to the phylum bacteria, 99.9% reads were assigned to order, 99.2% to family and 94.4% to genera (Table S2). For the ITS reads assigned to fungi, the proportions were 97.3%, 70.8% and 24.4%, and for plant ITS2 100%, 100% and 98.3%, respectively. Thus, we used the taxonomic level of family for analyses, on top of ZOTU level. Yet, the taxonomic assignments to genera for bacterial and fungal data are shown to describe the communities (Tables S4 and S5).

For 16S, for the flower samples the average read number per sample was very low 2909 (SD ±3929, Table S3). For honey and bee samples the average read numbers were nearly ten-fold in comparison to the flower samples, 16424 (SD ±8957) and 24728 (SD ±7375), respectively. For fungal ITS, again for the flower samples the number of reads on average was lower than for the honey and bee samples, 5156 (SD ±4666), 27889 (SD ±15836) and 10290 (SD ±12352), respectively. For the plant ITS2, for honey samples only, the samples had on average 25950 (SD ± 11229) reads.

The bacterial genera, which the five ubiquitous honey bee gut microbial species clusters belong to (*Lactobacillus* Firm-4 and Firm-5, *Snodgrassella alvi*, *Gilliamella apicola* and *Bifidobacterium* spp [17]), were found in all honey bee samples, as expected. They were found in a far smaller proportion of honey samples and nearly not at all in flower samples. The bacterial family Erwiniaceae, which includes *Erwinia,* a plant pathogen dispersed by honey bees [18], was the most frequently found bacterial family among flower samples. It was also found in most honey samples, yet only in 5.1% of honey bee samples (Table S5). Among fungi, the family Metschnikowiaceae and the genus *Metschnikowia*, a yeast able to grow in nectar and to change the nectar properties to attract more flower visitors [19], was the most shared fungal taxon among the sample types. Other fungal families and genera were mostly shared only by two sample types, among these being the families Ascosphaeraceae and Aspergillaceae, to which the common fungal honey bee pathogens *Ascosphaera apis* and *Aspergillus niger* belong to [20, 21], were common both in honey bee and honey samples, but not detected in the flower samples (Table S6).

Table S1. List of flowering plants sampled, with the total number of samples per species as well the the number of samples with data for bacteria (16S gene region) and fungi (ITS gene region).

|  |  | samples | Data for |  |
| --- | --- | --- | --- | --- |
| Family | Species | total | 16S | ITS |
| Apiaceae | | 8 | 8 | 7 |
|  | *Angelica sylvestris* | 4 | 4 | 4 |
|  | *Antriscus sylvestris* | 4 | 4 | 3 |
| Asteraceae | | 34 | 31 | 34 |
|  | *Achillea millefolium* | 3 | 3 | 3 |
|  | *Achillea ptarmica* | 4 | 4 | 4 |
|  | *Artemisia vulgaris* | 1 | 1 | 1 |
|  | *Bidens tripartita* | 1 | 1 | 1 |
|  | *Cirsium arvense* | 6 | 6 | 6 |
|  | *Cirsium heterophyllum* | 1 | 1 | 1 |
|  | *Cirsium palustre* | 3 | 3 | 3 |
|  | *Hieracium umbellatum* | 3 | 3 | 3 |
|  | *Leucanthemum vulgare* | 2 | 2 | 2 |
|  | *Scorzoneroides autumnalis* | 5 | 4 | 5 |
|  | *Senecio sylvaticus* | 1 | 0 | 1 |
|  | *Solidago virgaurea* | 2 | 1 | 2 |
|  | *Tripleurospermum inodorum* | 2 | 2 | 2 |
| Balsaminaceae | | 1 | 1 | 1 |
|  | *Impatiens glandulifera* | 1 | 1 | 1 |
| Campanulaceae | | 6 | 4 | 6 |
|  | *Campanula patula* | 1 | 0 | 1 |
|  | *Campanula rotundifolia* | 5 | 4 | 5 |
| Caryophyllaceae | | 3 | 0 | 0 |
|  | *Stellaria longifolia* | 1 | 0 | 0 |
|  | *Stellaria sp.* | 2 | 0 | 0 |
| Ericaceae | | 13 | 10 | 13 |
|  | *Calluna vulgaris* | 3 | 3 | 3 |
|  | *Ledum palustre* | 2 | 1 | 2 |
|  | *Vaccinium myrtillus* | 1 | 0 | 1 |
|  | *Vaccinium uliginosum* | 2 | 2 | 2 |
|  | *Vaccinium vitis-idaea* | 5 | 4 | 5 |
| Fabaceae | | 11 | 10 | 11 |
|  | *Ervilia sylvatica* | 1 | 1 | 1 |
|  | *Lathyrus pratensis* | 1 | 1 | 1 |
|  | *Trifolium pratense* | 6 | 6 | 6 |
|  | *Trifolium repens* | 1 | 1 | 1 |
|  | *Vicia cracca* | 2 | 1 | 2 |
| Geraniaceae | | 1 | 1 | 1 |
|  | *Geranium sylvaticum* | 1 | 1 | 1 |
| Hypericaceae | | 1 | 1 | 1 |
|  | *Hypericum maculatum* | 1 | 1 | 1 |
| Lamiaceae | | 5 | 5 | 5 |
|  | *Galeopsis bifida* | 1 | 1 | 1 |
|  | *Galeopsis speciosa* | 3 | 3 | 3 |
|  | *Lamium album* | 1 | 1 | 1 |
| Linaceae | | 1 | 0 | 0 |
|  | *Linum usitatissium* | 1 | 0 | 0 |
| Onagraceae | | 4 | 4 | 4 |
|  | *Epilobium angustifolium* | 4 | 4 | 4 |
| Orobanchaceae | | 12 | 11 | 12 |
|  | *Euphrasia sp.* | 2 | 2 | 2 |
|  | *Melampyrum pratense* | 7 | 6 | 7 |
|  | *Melampyrum sylvaticum* | 3 | 3 | 3 |
| Plantaginaceae | | 5 | 2 | 4 |
|  | *Linaria vulgaris* | 1 | 1 | 1 |
|  | *Veronica chamaedrys* | 4 | 1 | 3 |
| Polygonaceae | | 1 | 1 | 1 |
|  | *Polygonum aviculare* | 1 | 1 | 1 |
| Primulaceae | | 9 | 5 | 9 |
|  | *Lysimachia europaea* | 7 | 3 | 7 |
|  | *Lysimachia vulgaris* | 2 | 2 | 2 |
| Ranunculaceae | | 6 | 4 | 5 |
|  | *Ranunculus acris* | 6 | 4 | 5 |
| Rosaceae | | 16 | 6 | 12 |
|  | *Comarum palustre* | 1 | 0 | 0 |
|  | *Filipendula ulmaria* | 4 | 4 | 4 |
|  | *Fragaria vesca* | 2 | 0 | 1 |
|  | *Potentilla argentea* | 1 | 0 | 0 |
|  | *Potentilla erecta* | 6 | 2 | 6 |
|  | *Sorbus aucuparia* | 2 | 0 | 1 |
| Rubiaceae | | 3 | 2 | 3 |
|  | *Galium album* | 2 | 1 | 2 |
|  | *Galium verum* | 1 | 1 | 1 |
| Solanaceae | | 1 | 1 | 1 |
|  | *Solanum tuberosum* | 1 | 1 | 1 |
| Violaceae | | 3 | 3 | 3 |
|  | *Viola arvensis* | 2 | 2 | 2 |
|  | *Viola canina* | 1 | 1 | 1 |

Table S2. Number of reads after different steps of the bioinformatics processing for the three gene regions, with the number of reads assigned to different taxonomic levels.

| Reads | 16S bacteria | | ITS fungi | | ITS2 plant | |
| --- | --- | --- | --- | --- | --- | --- |
| Original | 9796332 |  | 5760195 |  | 3955084 |  |
| Truncated, merged and quality-filtered | 9258549 | 94.5%% | 4852590 | 84.2%% | 2755780 | 69.7%% |
| With adapters and passing filters | 9256026 | 100.0%% | 4849933 | 99.9%% | 2753108 | 99.9%% |
| Denoised to ZOTUs | 9021522 | 97.5%% | 4802172 | 99.0%% | 2677504 | 97.3%% |
| After filtering and assigned to target phylum | 4369359 | 48.4%% | 4444922 | 92.6%% | 2569122 | 96.0%% |
| Assigned to order, of assigned to phylum | 4366314 | 99.9%% | 4326615 | 97.3%% | 2568855 | 100.0%% |
| Assigned to family, of assigned to phylum | 4332504 | 99.2%% | 3146414 | 70.8%% | 2568855 | 100.0%% |
| Assigned to genus, of assigned to phylum | 4123473 | 94.4%% | 1086639 | 24.4%% | 2524808 | 98.3%% |
| Assigned to species, of assigned to phylum |  |  | 275950 | 6.2%% | 411527 | 16.0%% |
| Number of ZOTUs | 394 |  | 995 |  | 339 |  |
| Number of samples with data | 307 |  | 329 |  | 99 |  |
| Average number of reads per sample | 14232 |  | 13510 |  | 25951 |  |

Table S3. Number of samples with data for bacteria, fungi and plants, for the three different sample types of bees, honey and flowers. The average number of reads per sample and the standard deviation is given, based on all the samples with sequence reads for the concerned gene region.

| sample type/ reads | 16S bacteria | |  | ITS fungi | |  | ITS2 plant | |  |
| --- | --- | --- | --- | --- | --- | --- | --- | --- | --- |
|  | samples | mean | (SD ±) | samples | mean | (SD ±) | samples | mean | (SD ±) |
| Bees | 98 | 24728 | 7375 | 97 | 10290 | 12352 |  |  |  |
| Honey | 99 | 16424 | 8957 | 99 | 27889 | 15836 | 99 | 25950 | 11229 |
| Flower | 110 | 2909 | 3929 | 133 | 5156 | 4666 |  |  |  |
| All | 307 | 14232 | 11464 | 329 | 16510 | 14924 | 99 | 25950 | 11229 |

Table S4. The frequency of occurrence (FOO) of bacterial families and genera detected in the different sample types.

|  | Sample type | Bee | Honey | Flower |
| --- | --- | --- | --- | --- |
|  | Number of samples | 98 | 99 | 114 |
| Family | Genus |  |  |  |
| Acetobacteraceae | | 99.0 | 94.9 | 0.9 |
|  | *Acidisoma* |  | 3.0 |  |
|  | *Asaia* |  | 4.0 |  |
|  | *Bombella* | 4.1 | 90.9 |  |
|  | *Commensalibacter* | 13.3 | 2.0 |  |
|  | *Neokomagataea* |  | 7.1 |  |
|  | *Nguyenibacter* |  | 5.1 |  |
|  | *Tanticharoenia* |  | 7.1 |  |
| Aeromonadaceae | | 1.0 | 1.0 |  |
|  | *Aeromonas* | 1.0 |  |  |
|  | *Oceanisphaera* |  | 1.0 |  |
| Alcaligenaceae | |  | 2.0 | 0.9 |
|  | *Achromobacter* |  | 1.0 |  |
| Anaplasmataceae | |  |  | 3.5 |
| Arcobacteraceae | |  | 1.0 |  |
|  | *Aliarcobacter* |  | 1.0 |  |
| Aurantimonadaceae | |  |  | 6.1 |
|  | *Aureimonas* |  |  | 5.3 |
| Azospirillaceae | |  | 11.1 |  |
|  | *Azospirillum* |  | 10.1 |  |
| Bacillaceae | |  | 1.0 |  |
|  | *Bacillus* |  | 1.0 |  |
| Bacillales_Incertae_Sedis_XII | |  |  | 0.9 |
|  | *Exiguobacterium* |  |  | 0.9 |
| Bartonellaceae | | 66.3 | 8.1 |  |
|  | *Bartonella* | 66.3 | 8.1 |  |
| Beijerinckiaceae | |  | 1.0 |  |
| Bifidobacteriaceae | | 100.0 | 17.2 |  |
|  | *Bifidobacterium* | 100.0 | 17.2 |  |
| Burkholderiaceae | |  | 7.1 |  |
|  | *Paraburkholderia* |  | 3.0 |  |
| Carnobacteriaceae | |  | 6.1 |  |
|  | *Carnobacterium* |  | 6.1 |  |
| Caulobacteraceae | |  | 1.0 | 0.9 |
|  | *Brevundimonas* |  | 1.0 | 0.9 |
|  | *Caulobacter* |  | 1.0 |  |
| Comamonadaceae | |  | 8.1 | 7.0 |
|  | *Acidovorax* |  | 1.0 |  |
|  | *Comamonas* |  | 2.0 |  |
|  | *Delftia* |  | 1.0 |  |
|  | *Polaromonas* |  | 2.0 |  |
|  | *Ramlibacter* |  |  | 3.5 |
|  | *Roseateles* |  |  | 0.9 |
| Coxiellaceae | | 1.0 |  |  |
|  | *Diplorickettsia* | 1.0 |  |  |
| Cryomorphaceae | |  | 1.0 |  |
|  | *Fluviicola* |  | 1.0 |  |
| Cytophagaceae | |  | 3.0 |  |
|  | *Spirosoma* |  | 3.0 |  |
| Dysgonomonadaceae | |  |  | 1.8 |
|  | *Dysgonomonas* |  |  | 1.8 |
| Enterobacteriaceae | | 16.3 | 75.8 | 51.8 |
|  | *Buttiauxella* |  | 1.0 |  |
|  | *Kosakonia* | 3.1 |  | 1.8 |
|  | *Pseudescherichia* | 10.2 | 12.1 | 3.5 |
|  | *Raoultella* | 1.0 | 73.7 | 32.5 |
| Enterococcaceae | |  | 6.1 | 0.9 |
|  | *Enterococcus* |  | 2.0 | 0.9 |
|  | *Vagococcus* |  | 3.0 |  |
| Entomoplasmataceae | |  | 32.3 | 6.1 |
|  | *Mesoplasma* |  | 32.3 | 6.1 |
| Erwiniaceae | | 5.1 | 83.8 | 55.3 |
|  | *Buchnera* |  |  | 5.3 |
|  | *Erwinia* | 1.0 | 67.7 | 14.9 |
|  | *Pantoea* | 3.1 | 17.2 | 35.1 |
|  | *Tatumella* | 1.0 | 56.6 | 11.4 |
| Erythrobacteraceae | |  | 5.1 | 0.9 |
|  | *Novosphingobium* |  | 5.1 | 0.9 |
| Flavobacteriaceae | |  | 2.0 |  |
|  | *Flavobacterium* |  | 2.0 |  |
| Hafniaceae | | 32.7 | 4.0 | 6.1 |
|  | *Hafnia* | 32.7 | 4.0 | 6.1 |
| Halomonadaceae | |  | 4.0 | 1.8 |
|  | *Zymobacter* |  | 4.0 | 0.9 |
| Hymenobacteraceae | |  | 1.0 | 4.4 |
|  | *Hymenobacter* |  | 1.0 | 4.4 |
| Jonesiaceae | |  | 1.0 |  |
|  | *Sanguibacter* |  | 1.0 |  |
| Kineosporiaceae | |  | 1.0 |  |
| Lactobacillaceae | | 100.0 | 93.9 | 1.8 |
|  | *Apilactobacillus* | 1.0 | 83.8 | 0.9 |
|  | *Bombilactobacillus* | 100.0 | 16.2 |  |
|  | *Fructobacillus* | 2.0 | 30.3 |  |
|  | *Holzapfelia* |  | 11.1 | 0.9 |
|  | *Lactiplantibacillus* |  |  |  |
|  | *Lactobacillus* | 100.0 | 33.3 |  |
|  | *Latilactobacillus* |  | 11.1 |  |
|  | *Leuconostoc* |  | 1.0 |  |
| Listeriaceae | |  | 1.0 |  |
|  | *Brochothrix* |  | 1.0 |  |
| Methylobacteriaceae | |  | 12.1 | 12.3 |
|  | *Methylobacterium* |  | 3.0 | 10.5 |
| Methylophilaceae | |  | 1.0 |  |
|  | *Methylophilus* |  | 1.0 |  |
| Microbacteriaceae | |  | 4.0 | 5.3 |
|  | *Rathayibacter* |  |  | 0.9 |
| Micrococcaceae | |  | 1.0 | 0.9 |
|  | *Arthrobacter* |  | 1.0 |  |
|  | *Paeniglutamicibacter* |  |  | 0.9 |
| Moraxellaceae | | 4.1 | 40.4 | 11.4 |
|  | *Acinetobacter* | 4.1 | 39.4 | 11.4 |
|  | *Alkanindiges* |  |  |  |
| Morganellaceae | | 12.2 | 6.1 | 4.4 |
|  | *Arsenophonus* |  | 5.1 |  |
|  | *Morganella* | 3.1 |  | 2.6 |
|  | *Proteus* | 6.1 | 1.0 |  |
|  | *Providencia* | 7.1 | 1.0 | 3.5 |
| NA |  |  |  |  |
|  | *Ignatzschineria* |  | 1.0 |  |
| Neisseriaceae | | 100.0 | 8.1 |  |
|  | *Snodgrassella* | 100.0 | 8.1 |  |
| Oceanospirillaceae | |  | 1.0 |  |
|  | *Marinospirillum* |  | 1.0 |  |
| Orbaceae | | 100.0 | 70.7 | 5.3 |
|  | *Frischella* | 95.9 | 25.3 | 0.9 |
|  | *Gilliamella* | 100.0 | 31.3 | 0.9 |
|  | *Orbus* |  | 8.1 | 3.5 |
| Oxalobacteraceae | |  | 2.0 | 21.1 |
|  | *Duganella* |  |  | 4.4 |
|  | *Massilia* |  | 1.0 | 18.4 |
| Paenibacillaceae | |  |  | 2.6 |
|  | *Paenibacillus* |  |  | 2.6 |
| Paludibacteraceae | |  | 2.0 |  |
|  | *Paludibacter* |  | 2.0 |  |
| Pasteurellaceae | |  |  | 0.9 |
|  | *Haemophilus* |  |  | 0.9 |
| Pectobacteriaceae | |  | 6.1 |  |
|  | *Pectobacterium* |  | 1.0 |  |
|  | *Sodalis* |  | 5.1 |  |
| Planococcaceae | |  | 1.0 |  |
|  | *Sporosarcina* |  | 1.0 |  |
| Pseudomonadaceae | | 24.5 | 25.3 | 46.5 |
|  | *Pseudomonas* | 23.5 | 24.2 | 46.5 |
| Rhizobiaceae | |  | 5.1 | 3.5 |
|  | *Neorhizobium* |  |  | 0.9 |
|  | *Rhizobium* |  | 5.1 | 3.5 |
| Rhodanobacteraceae | |  | 9.1 | 1.8 |
|  | *Dyella* |  | 1.0 |  |
|  | *Luteibacter* |  | 8.1 | 1.8 |
|  | *Rhodanobacter* |  | 2.0 |  |
| Rhodospirillaceae | |  | 2.0 |  |
|  | *Magnetospirillum* |  | 2.0 |  |
| Rickettsiaceae | |  |  | 4.4 |
|  | *Rickettsia* |  |  | 4.4 |
| Sphingobacteriaceae | |  | 10.1 | 3.5 |
|  | *Mucilaginibacter* |  | 1.0 |  |
|  | *Pedobacter* |  | 9.1 | 3.5 |
|  | *Sphingobacterium* |  | 1.0 | 0.9 |
| Sphingomonadaceae | |  | 4.0 | 34.2 |
|  | *Sphingobium* |  | 1.0 | 0.0 |
|  | *Sphingomonas* |  | 4.0 | 34.2 |
| Spiroplasmataceae | | 2.0 | 24.2 | 4.4 |
|  | *Spiroplasma* | 2.0 | 24.2 | 4.4 |
| Streptococcaceae | |  | 13.1 | 0.9 |
|  | *Lactococcus* |  | 13.1 | 0.9 |
| Verrucomicrobiaceae | |  | 3.0 |  |
|  | *Prosthecobacter* |  | 3.0 |  |
| Weeksellaceae | | 19.4 |  | 1.8 |
|  | *Apibacter* | 19.4 |  |  |
|  | *Chryseobacterium* |  |  | 1.8 |
| Xanthomonadaceae | |  | 2.0 | 1.8 |
|  | *Lysobacter* |  | 1.0 |  |
|  | *Stenotrophomonas* |  | 1.0 | 0.9 |
|  | *Xanthomonas* |  |  | 1.8 |
| Yersiniaceae | | 6.1 | 47.5 | 3.5 |
|  | *Serratia* | 3.1 | 14.1 | 1.8 |
|  | *Yersinia* | 1.0 | 5.1 | 1.8 |

Table S5. The frequency of occurrence (FOO) of fungal families and genera detected in the different sample types.

|  | Sample type | Bee | Honey | Flower |
| --- | --- | --- | --- | --- |
|  | Number of samples | 97 | 99 | 133 |
| Family | Genus |  |  |  |
| Ascosphaeraceae | | 53.6 | 73.7 |  |
| Aspergillaceae | | 32.0 | 43.4 |  |
|  | *Aspergillus* | 17.5 | 29.3 |  |
|  | *Penicillium* | 26.8 | 32.3 |  |
| Aureobasidiaceae | | 4.1 | 44.4 | 65.4 |
|  | *Aureobasidium* | 2.1 | 22.2 | 30.8 |
|  | *Kabatiella* | 1.0 | 29.3 | 62.4 |
| Bondarzewiaceae | |  |  | 0.8 |
|  | *Heterobasidion* |  |  | 0.8 |
| Botryobasidiaceae | |  |  | 2.3 |
|  | *Botryobasidium* |  |  | 2.3 |
| Botryosphaeriaceae | |  |  | 2.3 |
|  | *Botryosphaeria* |  |  | 2.3 |
| Bulleraceae | |  |  | 3.8 |
|  | *Bullera* |  |  | 0.8 |
|  | *Genolevuria* |  |  | 2.3 |
| Bulleribasidiaceae | | 1.0 | 4.0 | 56.4 |
|  | *Dioszegia* |  |  | 0.8 |
|  | *Vishniacozyma* | 1.0 | 4.0 | 56.4 |
| Cephalothecaceae | | 1.0 |  |  |
|  | *Phialemonium* | 1.0 |  |  |
| Ceratobasidiaceae | |  |  | 1.5 |
| Cladosporiaceae | | 41.2 | 40.4 | 74.4 |
|  | *Cladosporium* | 7.2 | 5.1 |  |
|  | *Verrucocladosporium* |  |  |  |
| Clavicipitaceae | | 2.1 | 5.1 | 9.8 |
|  | *Claviceps* | 1.0 | 5.1 | 4.5 |
| Coleosporiaceae | |  | 7.1 | 0.8 |
|  | *Chrysomyxa* |  | 7.1 |  |
|  | *Coleosporium* |  |  | 0.8 |
| Coniochaetaceae | | 2.1 | 5.1 |  |
|  | *Coniochaeta* | 2.1 | 5.1 |  |
| Cordycipitaceae | | 7.2 | 2.0 |  |
|  | *Simplicillium* | 2.1 | 1.0 |  |
| Cystobasidiaceae | |  | 1.0 |  |
|  | *Cystobasidium* |  | 1.0 |  |
| Cystofilobasidiaceae | |  |  | 4.5 |
|  | *Cystofilobasidium* |  |  | 4.5 |
| Debaryomycetaceae | | 2.1 | 6.1 |  |
|  | *Debaryomyces* | 2.1 | 6.1 |  |
| Dermateaceae | | 1.0 |  | 9.0 |
|  | *Calloria* |  |  | 6.8 |
|  | *Naevala* | 1.0 |  | 1.5 |
| Didymellaceae | |  |  | 14.3 |
|  | *Neoascochyta* |  |  | 3.8 |
| Didymosphaeriaceae | |  |  | 3.0 |
| Dissoconiaceae | |  |  | 2.3 |
|  | *Dissoconium* |  |  | 2.3 |
| Dothioraceae | | 6.2 | 17.2 | 7.5 |
|  | *Perusta* | 4.1 | 11.1 | 3.0 |
| Entylomataceae | |  |  | 1.5 |
|  | *Entyloma* |  |  | 1.5 |
| Erysiphaceae | | 9.3 | 8.1 | 25.6 |
|  | *Blumeria* | 2.1 |  | 1.5 |
|  | *Erysiphe* | 5.2 | 6.1 | 3.8 |
|  | *Golovinomyces* |  |  | 7.5 |
|  | *Neoerysiphe* | 2.1 | 5.1 | 2.3 |
|  | *Podosphaera* | 1.0 | 1.0 | 15.8 |
| Exobasidiaceae | |  | 1.0 | 0.8 |
|  | *Exobasidium* |  | 1.0 | 0.8 |
| Filobasidiaceae | |  |  | 14.3 |
|  | *Filobasidium* |  |  | 14.3 |
|  | *Naganishia* |  |  | 0.8 |
| Glomerellaceae | |  |  | 2.3 |
|  | *Colletotrichum* |  |  | 2.3 |
| Haematommataceae | | 2.1 | 2.0 |  |
|  | *Haematomma* | 2.1 | 2.0 |  |
| Helicobasidiaceae | | 1.0 |  |  |
|  |  |  |  | 1.5 |
| Helotiaceae | *Godronia* |  |  | 0.8 |
|  | *Meliniomyces* |  |  | 0.8 |
| Helotiales_fam_Incertae_sedis | |  |  | 2.3 |
| Herpotrichiellaceae | |  | 2.0 | 1.5 |
| Holtermanniales_fam_Incertae_sedis | |  |  | 6.8 |
|  | *Holtermanniella* |  |  | 6.8 |
| Hyaloscyphaceae | |  | 1.0 | 0.8 |
|  | *Hyaloscypha* |  | 1.0 |  |
| Hymenochaetales_fam_Incertae_sedis | |  |  | 0.8 |
|  | *Resinicium* |  |  | 0.8 |
| Hypocreales_fam_Incertae_sedis | | 2.1 |  |  |
|  | *Acremonium* | 1.0 |  |  |
|  | *Sarocladium* | 1.0 |  |  |
| Lecanoraceae | |  | 3.0 | 0.8 |
|  | *Scoliciosporum* |  | 1.0 |  |
| Leptosphaeriaceae | |  |  | 4.5 |
|  | *Ampelomyces* |  |  | 0.8 |
|  | *Plenodomus* |  |  | 0.8 |
| Leucosporidiaceae | |  | 1.0 | 7.5 |
|  | *Leucosporidium* |  | 1.0 | 7.5 |
| Malasseziaceae | |  |  | 0.8 |
| Melanommataceae | |  | 1.0 | 3.8 |
|  | *Alpinaria* |  | 1.0 | 0.8 |
| Metschnikowiaceae | | 60.8 | 88.9 | 29.3 |
|  | *Kodamaea* | 1.0 |  |  |
|  | *Metschnikowia* | 60.8 | 88.9 | 29.3 |
| Microbotryomycetes_fam_Incertae_sedis | |  |  | 2.3 |
|  | *Curvibasidium* |  |  | 2.3 |
| Microdochiaceae | |  |  | 1.5 |
|  | *Microdochium* |  |  | 1.5 |
| Mrakiaceae | |  |  | 20.3 |
|  | *Itersonilia* |  |  | 19.5 |
|  | *Mrakia* |  |  | 3.0 |
| Mucoraceae | | 32.0 | 3.0 | 1.5 |
|  | *Mucor* | 27.8 | 2.0 | 1.5 |
| Mycocaliciaceae | |  | 1.0 |  |
|  | *Chaenothecopsis* |  | 1.0 |  |
| Mycosphaerellaceae | | 9.3 | 3.0 | 49.6 |
|  | *Ramularia* | 9.3 | 3.0 | 40.6 |
|  | *Sphaerulina* |  |  | 0.8 |
|  | *Zymoseptoria* | 1.0 |  | 15.8 |
| Myxotrichaceae | | 21.6 | 17.2 |  |
|  | *Oidiodendron* | 18.6 | 14.1 |  |
| Nectriaceae | |  |  | 17.3 |
|  | *Cosmospora* |  |  | 0.8 |
|  | *Fusarium* |  |  | 5.3 |
|  | *Gibberella* |  |  | 12.0 |
| Orbiliaceae | |  |  | 0.8 |
| Parmeliaceae | |  | 6.1 | 6.0 |
|  | *Hypogymnia* |  | 5.1 | 6.0 |
|  | *Platismatia* |  | 1.0 |  |
| Pezizomycotina_fam_Incertae_sedis | |  | 1.0 | 2.3 |
|  | *Ciliophora* |  | 1.0 | 2.3 |
| Phacidiaceae | |  |  | 6.8 |
|  | *Phacidium* |  |  | 6.0 |
| Phaeococcomycetaceae | |  | 5.1 |  |
|  | *Phaeococcomyces* |  | 5.1 |  |
| Phaeosphaeriaceae | | 2.1 | 1.0 | 15.0 |
|  | *Muriphaeosphaeria* |  |  | 0.8 |
|  | *Sclerostagonospora* |  |  | 0.8 |
|  | *Septoriella* |  |  | 2.3 |
| Phaffomycetaceae | |  | 1.0 |  |
|  | *Wickerhamomyces* |  | 1.0 |  |
| Plectosphaerellaceae | |  | 1.0 | 0.8 |
|  | *Plectosphaerella* |  | 1.0 | 0.8 |
| Pleosporaceae | | 44.3 | 51.5 | 69.9 |
|  | *Alternaria* | 19.6 | 11.1 | 51.1 |
|  | *Bipolaris* | 30.9 | 47.5 | 4.5 |
|  | *Briansuttonomyces* | 1.0 | 1.0 | 9.8 |
|  | *Stemphylium* |  | 1.0 | 2.3 |
| Polyporaceae | |  |  | 1.5 |
| Pseudeurotiaceae | | 5.2 | 24.2 | 0.8 |
|  | *Bettsia* | 2.1 | 23.2 |  |
| Pucciniastraceae | |  | 13.1 | 9.0 |
|  | *Pucciniastrum* |  |  | 0.8 |
|  | *Thekopsora* |  | 13.1 | 8.3 |
| Rhynchogastremataceae | |  |  | 7.5 |
|  | *Papiliotrema* |  |  | 1.5 |
| Rhytismataceae | | 1.0 |  | 3.0 |
|  | *Lophodermium* | 1.0 |  | 2.3 |
|  | *Tryblidiopsis* |  |  | 0.8 |
| Saccharomycetaceae | |  | 5.1 |  |
|  | *Citeromyces* |  | 5.1 |  |
| Saccharomycetales_fam_Incertae_sedis | | 26.8 | 39.4 | 0.8 |
|  | *Candida* | 26.8 | 36.4 | 0.8 |
|  | *Starmerella* |  | 5.1 |  |
| Sarcoscyphaceae | |  |  | 0.8 |
|  | *Desmazierella* |  |  | 0.8 |
| Sclerotiniaceae | | 5.2 | 7.1 | 19.5 |
|  | *Botryotinia* | 1.0 | 2.0 | 13.5 |
|  | *Monilinia* | 4.1 | 6.1 | 4.5 |
| Sirobasidiaceae | |  |  | 0.8 |
| Sporidiobolaceae | |  |  | 22.6 |
| Sporidiobolaceae | *Rhodotorula* |  |  | 3.8 |
|  | *Sporobolomyces* |  |  | 21.8 |
| Sporocadaceae | |  |  | 1.5 |
| Stachybotryaceae | |  | 1.0 |  |
| Stictidaceae | |  | 1.0 |  |
|  | *Cryptodiscus* |  | 1.0 |  |
| Symmetrosporaceae | |  |  | 0.8 |
|  | *Symmetrospora* |  |  | 0.8 |
| Sympoventuriaceae | | 1.0 |  |  |
|  | *Fusicladium* | 1.0 |  |  |
| Teloschistaceae | |  |  | 0.8 |
|  | *Polycauliona* |  |  | 0.8 |
| Teratosphaeriaceae | | 1.0 | 3.0 | 15.8 |
|  | *Apenidiella* |  |  | 4.5 |
|  | *Capnobotryella* |  |  | 1.5 |
|  | *Elasticomyces* | 1.0 | 3.0 |  |
|  | *Lapidomyces* |  |  | 3.8 |
|  | *Neophaeothecoidea* |  |  | 3.8 |
| Thelebolaceae | |  |  | 0.8 |
|  | *Thelebolus* |  |  | 0.8 |
| Tremellaceae | |  |  | 0.8 |
|  | *Cryptococcus* |  |  | 0.8 |
| Trichocomaceae | | 8.2 | 7.1 |  |
|  | *Talaromyces* | 8.2 | 7.1 |  |
| Valsaceae |  | 1.0 |  |  |
| Venturiaceae | | 1.0 | 2.0 | 3.0 |
|  | *Venturia* |  | 2.0 | 0.8 |
| Wallemiaceae | |  | 1.0 |  |
|  | *Wallemia* |  | 1.0 |  |


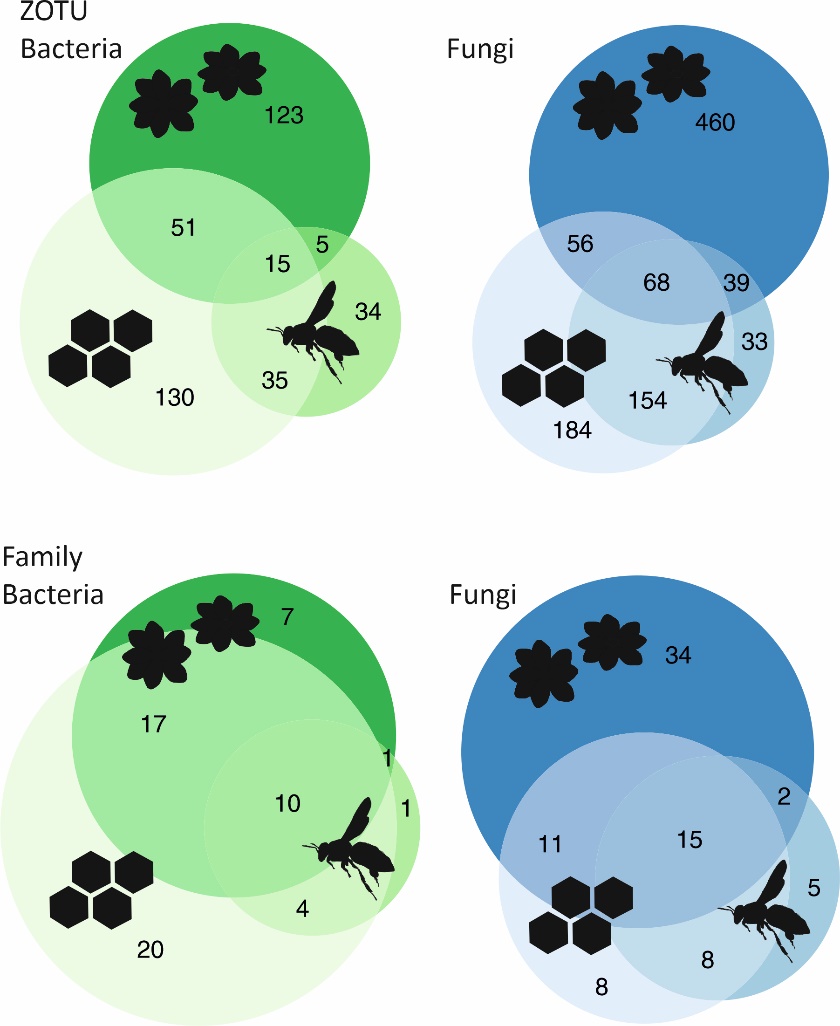


Figure S1. Euler diagrams showing the number of shared bacterial and fungal ZOTUs and families between bee, honey and flower samples, shown with the symbols, with all samples and all taxa included. Bacterial and fungal ZOTUs and families are shown.


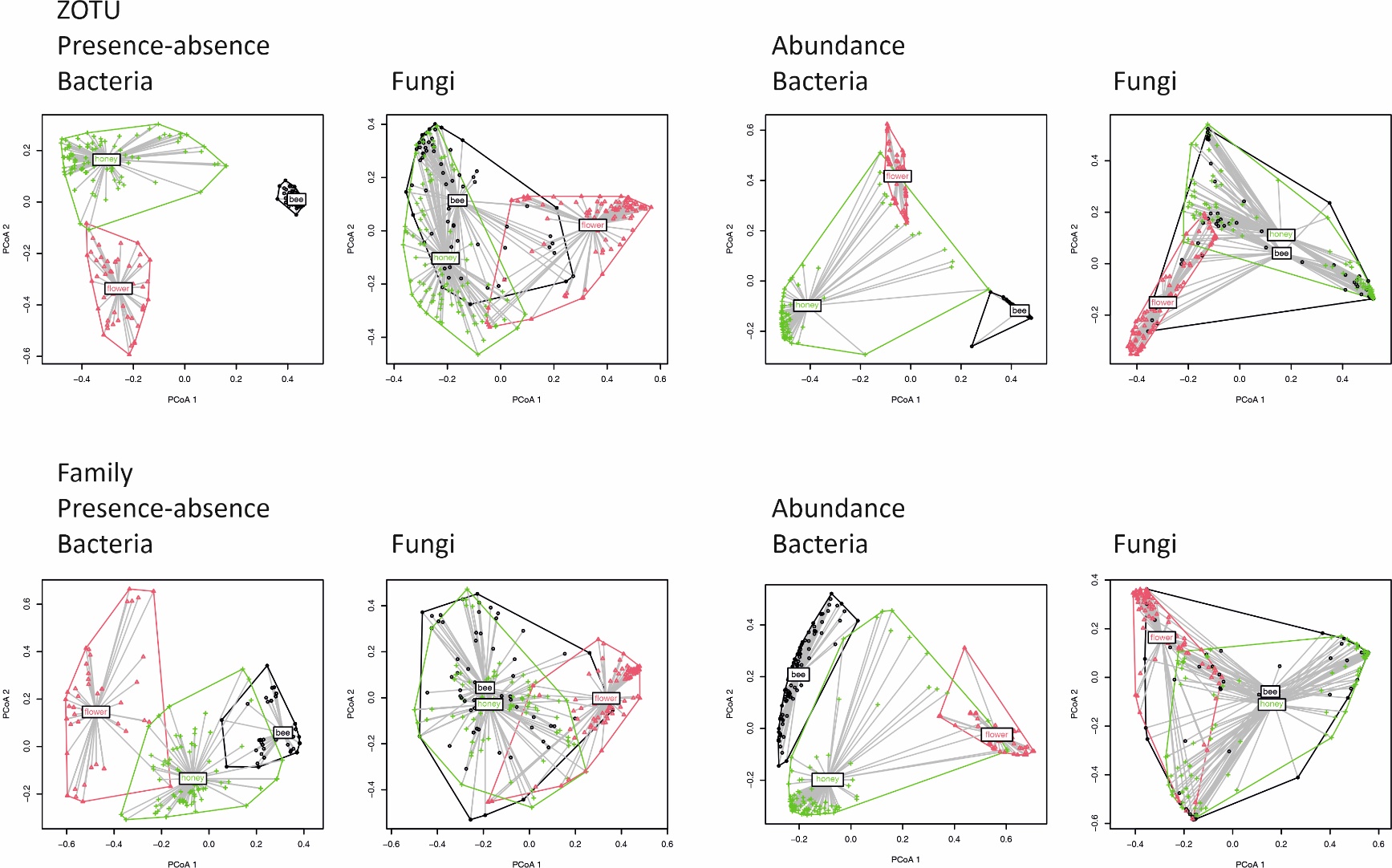


Figure S2. Beta-diversity of bacterial and fungal communities in honey, bee, and flower samples based on principal coordinates analyses (axes 1 & 2) for the taxonomic level of ZOTUs and families (first and second line) for both presence-absence and relative read abundance data (first and second column). Samples with less than 2000 reads and taxa with less than three occurrences are omitted from the analyses. The panels with ZOTU and presence-absence data are identical to the ones shown in Fig. 2.

Table S6. Results of the multivariate homogeneity of group dispersion analyses (i.e., beta-diversity) and the principal component analyses, for both the presence-absence (PA) and relative read abundance data (Abundance), as shown in Figs 2 and S2.

|  |  | ZOTU |  |  |  | Family |  |  |  |
| --- | --- | --- | --- | --- | --- | --- | --- | --- | --- |
|  |  | PA |  | Abundance | | PA |  | Abundance | |
|  |  | Bacteria | Fungi | Bacteria | Fungi | Bacteria | Fungi | Bacteria | Fungi |
| Average distance to centroid | | |  |  |  |  |  |  |  |
|  | bee | 0.168 | 0.572 | 0.293 | 0.573 | 0.123 | 0.4373 | 0.214 | 0.5414 |
|  | flower | 0.589 | 0.527 | 0.606 | 0.553 | 0.440 | 0.4006 | 0.519 | 0.4984 |
|  | honey | 0.466 | 0.500 | 0.307 | 0.560 | 0.311 | 0.3925 | 0.280 | 0.5288 |
| Eigenvalues for PCoA axes | |  |  |  |  |  |  |  |  |
|  | PCoA1 | 33.311 | 21.859 | 38.150 | 29.691 | 24.507 | 23.627 | 25.067 | 32.618 |
|  | PCoA2 | 9.616 | 11.282 | 14.411 | 13.474 | 8.826 | 10.274 | 14.489 | 19.821 |
|  | PCoA3 | 5.503 | 7.825 | 5.366 | 7.752 | 4.398 | 7.97 | 6.441 | 10.877 |
|  | PCoA4 | 3.855 | 6.347 | 3.421 | 6.047 | 3.874 | 5.955 | 4.777 | 6.703 |
|  | PCoA5 | 3.124 | 4.347 | 2.989 | 4.932 | 3.224 | 4.935 | 3.334 | 5.005 |
|  | PCoA6 | 2.332 | 4.324 | 2.502 | 3.458 | 2.935 | 4.792 | 2.457 | 3.312 |
|  | PCoA7 | 2.232 | 3.001 | 2.402 | 2.751 | 2.523 | 3.394 | 2.047 | 2.541 |
|  | PCoA8 | 2.181 | 2.337 | 2.247 | 2.700 | 2.283 | 3.157 | 1.661 | 2.363 |

Table S7. Results of the redundancy analyses for microbial communities of the different sample types, for the communities of ZOTUs based on presence-absence data, as well as the ANOVA results for the variance partitioned.

|  | Bacteria | | | | | | Fungi | | | |  |
| --- | --- | --- | --- | --- | --- | --- | --- | --- | --- | --- | --- |
|  | Df | variance | F | p-value | adjR2 |  | Df | variance | F | p-value | adjR2 |
| Bees |  |  |  |  |  |  |  |  |  |  |  |
| Model | 4 | 0.803 | 2.935 | **0.001** | 7.3%% |  | 4 | 3.547 | 4.840 | **0.001** | 18.3%% |
| Residual | 91 | 6.227 |  |  |  |  | 64 | 11.726 |  |  |  |
| Honey |  |  |  |  |  |  |  |  |  |  |  |
| Model | 4 | 2.178 | 5.307 | **0.001** | 15.0%% |  | 4 | 4.360 | 5.671 | **0.001** | 16.2%% |
| Residual | 90 | 9.233 |  |  |  |  | 90 | 17.298 |  |  |  |
| Flowers |  |  |  |  |  |  |  |  |  |  |  |
| Model | 4 | 0.897 | 1.357 | **0.035** | 2.8%% |  | 4 | 2.626 | 2.987 | **0.001** | 8.1%% |
| Residual | 44 | 7.276 |  |  |  |  | 84 | 18.4656 |  |  |  |
| Variables | Df | variance | F | p-value |  |  | Df | variance | F | p-value |  |
| Bees |  |  |  |  |  |  |  |  |  |  |  |
| month | 2 | 0.514 | 3.758 | **0.001** |  |  | 2 | 2.546 | 6.949 | **0.001** |  |
| region | 2 | 0.289 | 2.112 | **0.001** |  |  | 2 | 1.001 | 2.731 | **0.001** |  |
| Residual | 91 | 6.227 |  |  |  |  | 64 | 11.726 |  |  |  |
| Honey |  |  |  |  |  |  |  |  |  |  |  |
| month | 2 | 1.208 | 5.887 | **0.001** |  |  | 2 | 2.002 | 5.208 | **0.001** |  |
| region | 2 | 0.970 | 4.727 | **0.001** |  |  | 2 | 2.358 | 6.134 | **0.001** |  |
| Residual | 90 | 9.233 |  |  |  |  | 90 | 17.298 |  |  |  |
| Flowers |  |  |  |  |  |  |  |  |  |  |  |
| month | 2 | 0.422 | 1.277 | 0.123 |  |  | 2 | 1.984 | 4.513 | **0.001** |  |
| region | 2 | 0.475 | 1.437 | **0.023** |  |  | 2 | 0.642 | 1.460 | **0.015** |  |
| Residual | 44 | 7.276 |  |  |  |  | 84 | 18.466 |  |  |  |

Table S8. Results of the redundancy analyses for microbial communities of the different sample types, for the communities of families based on presence-absence data, as well as the ANOVA results for the variance partitioned.

|  | Bacteria | |  |  |  |  | Fungi | |  |  |  |
| --- | --- | --- | --- | --- | --- | --- | --- | --- | --- | --- | --- |
| Fractions | Df | adjR2 |  |  |  |  | Df | adjR2 |  |  |  |
| Bees |  |  |  |  |  |  |  |  |  |  |  |
| month | 2 | 15.3%% |  |  |  |  | 2 | 11.7%% |  |  |  |
| region | 2 | 1.5%% |  |  |  |  | 2 | 4.2%% |  |  |  |
| reads | 1 | 4.0%% |  |  |  |  | 1 | 1.7%% |  |  |  |
| residuals |  | 82.5%% |  |  |  |  |  | 81.5%% |  |  |  |
| Honey |  |  |  |  |  |  |  |  |  |  |  |
| month | 2 | 9.5%% |  |  |  |  | 2 | 7.6%% |  |  |  |
| region | 2 | 9.9%% |  |  |  |  | 2 | 10.6%% |  |  |  |
| reads | 1 | 4.5%% |  |  |  |  | 1 | 4.9%% |  |  |  |
| residuals |  |  |  |  |  |  |  | 77.6%% |  |  |  |
| Flowers |  | 79.9%% |  |  |  |  |  |  |  |  |  |
| month | 2 | 2.4%% |  |  |  |  | 2 | 6.7%% |  |  |  |
| region | 2 | 1.2%% |  |  |  |  | 2 | 0.5%% |  |  |  |
| reads | 1 | 0.5%% |  |  |  |  | 1 | 1.6%% |  |  |  |
| residuals |  | 95.2%% |  |  |  |  |  | 91.8%% |  |  |  |
|  | Df | variance | F | p-value | adjR2 |  | Df | variance | F | p-value | adjR2 |
| Bees |  |  |  |  |  |  |  |  |  |  |  |
| Model | 4 | 0.207 | 4.888 | **0.001** | 13.5% |  | 4 | 0.565 | 4.453 | **0.001** | 16.8% |
| Residual | 91 | 0.963 |  |  |  |  | 63 | 1.997 |  |  |  |
| Honey |  |  |  |  |  |  |  |  |  |  |  |
| Model | 4 | 0.656 | 5.593 | **0.001** | 15.6% |  | 4 | 0.632 | 6.306 | **0.001** | 17.5% |
| Residual | 90 | 2.639 |  |  |  |  | 90 | 2.254 |  |  |  |
| Flowers |  |  |  |  |  |  |  |  |  |  |  |
| Model | 4 | 0.300 | 1.549 | **0.021** | 4.4% |  | 4 | 0.447 | 2.594 | **0.001** | 6.6% |
| Residual | 44 | 2.131 |  |  |  |  | 84 | 3.616 |  |  |  |
| Variables | Df | variance | F | p-value |  |  | Df | variance | F | p-value |  |
| Bees |  |  |  |  |  |  |  |  |  |  |  |
| month | 2 | 0.158 | 7.450 | **0.001** |  |  | 2 | 0.378 | 5.969 | **0.001** |  |
| region | 2 | 0.049 | 2.326 | **0.005** |  |  | 2 | 0.186 | 2.938 | **0.001** |  |
| Residual | 91 | 0.963 |  |  |  |  | 63 | 1.997 |  |  |  |
| Honey |  |  |  |  |  |  |  |  |  |  |  |
| month | 2 | 0.394 | 6.715 | **0.001** |  |  | 2 | 0.269 | 5.378 | **0.001** |  |
| region | 2 | 0.262 | 4.471 | **0.001** |  |  | 2 | 0.362 | 7.234 | **0.001** |  |
| Residual | 90 | 2.639 |  |  |  |  | 90 | 2.254 |  |  |  |
| Flowers |  |  |  |  |  |  |  |  |  |  |  |
| month | 2 | 0.159 | 1.637 | **0.039** |  |  | 2 | 0.330 | 3.837 | **0.001** |  |
| region | 2 | 0.142 | 1.462 | 0.064 |  |  | 2 | 0.116 | 1.351 | 0.060 |  |
| Residual | 44 | 2.131 |  |  |  |  | 84 | 3.616 |  |  |  |

Table S9. Results of the redundancy analyses for the flower samples, considering the family of the flower sample in the model as well, for both microbial communities based on ZOTUs with presence-absence data, as well as the ANOVA results for the variance partitioned.

|  | Bacteria | | | | | | Fungi | | | |  |
| --- | --- | --- | --- | --- | --- | --- | --- | --- | --- | --- | --- |
| Fractions | Df | adjR2 |  |  |  |  | Df | adjR2 |  |  |  |
| Flowers |  |  |  |  |  |  |  |  |  |  |  |
| month | 2 | 1.0%% |  |  |  |  | 2 | 8.1%% |  |  |  |
| region | 2 | 1.8%% |  |  |  |  | 2 | 0.6%% |  |  |  |
| flower identity | 12 | 9.1%% |  |  |  |  | 16 | 11.0%% |  |  |  |
| reads | 1 | 1.9%% |  |  |  |  | 1 | 2.5%% |  |  |  |
| residuals |  | 89.9%% |  |  |  |  |  | 81.6%% |  |  |  |
|  | Df | variance | F | p-value | adjR2 |  | Df | variance | F | p-value | adjR2 |
| Flowers |  |  |  |  |  |  |  |  |  |  |  |
| Model | 16 | 3.180 | 1.274 | **0.024** | 8.2%% |  | 20 | 7.454 | 1.858 | **0.001** | 15.9%% |
| Residual | 32 | 4.992 |  |  |  |  | 68 | 13.638 |  |  |  |
| Variables | Df | variance | F | p-value |  |  | Df | variance | F | p-value |  |
| Flowers |  |  |  |  |  |  |  |  |  |  |  |
| month | 2 | 0.422 | 1.353 | 0.109 |  |  | 2 | 1.984 | 4.947 | **0.001** |  |
| region | 2 | 0.475 | 1.523 | **0.028** |  |  | 2 | 0.642 | 1.600 | **0.008** |  |
| flower identity | 12 | 2.283 | 1.220 | 0.066 |  |  | 16 | 4.827 | 1.504 | **0.001** |  |
| Residual | 32 | 4.992 |  |  |  |  | 68 | 13.638 |  |  |  |

Table S10. Results of the redundancy analyses for bees and honey samples, considering the relative proportions of eight most abundantly used flower families based on the honey samples, for each hive at each time point. The results of the redundancy analyses are shown for bacterial and fungal communities based on ZOTUs, with presence-absence data, as well as the ANOVA results for the variance partitioned.

|  | Bacteria | |  |  |  |  | Fungi | |  |  |  |
| --- | --- | --- | --- | --- | --- | --- | --- | --- | --- | --- | --- |
| Fractions | Df | adjR2 |  |  |  |  | Df | adjR2 |  |  |  |
| Bees |  |  |  |  |  |  |  |  |  |  |  |
| Plant families | 8 | 3.9%% |  |  |  |  | 8 | 8.8%% |  |  |  |
| Reads | 1 | 2.7%% |  |  |  |  | 1 | 0.4%% |  |  |  |
| Residuals |  | 94.5%% |  |  |  |  |  | 91.0%% |  |  |  |
| Honey |  |  |  |  |  |  |  |  |  |  |  |
| Plant families | 8 | 9.9%% |  |  |  |  | 8 | 8.7%% |  |  |  |
| Reads | 1 | 3.3%% |  |  |  |  | 1 | 2.3%% |  |  |  |
| Residuals |  | 87.2%% |  |  |  |  |  | 89.3%% |  |  |  |
|  | Df | variance | F | p-value | adjR2 |  | Df | variance | F | p-value | adjR2 |
| Bees |  |  |  |  |  |  |  |  |  |  |  |
| Model | 8 | 0.806 | 1.347 | **0.002** | 2.9%% |  | 8 | 3.114 | 1.752 | **0.001** | 8.6%% |
| Residual | 83 | 6.209 |  |  |  |  | 56 | 12.440 |  |  |  |
| Honey |  |  |  |  |  |  |  |  |  |  |  |
| Model | 8 | 2.003 | 2.288 | **0.001** | 9.6%% |  | 8 | 3.550 | 2.107 | **0.001** | 8.4%% |
| Residual | 86 | 9.409 |  |  |  |  | 86 | 18.109 |  |  |  |
| Variables | Df | variance | F | p-value |  |  | Df | variance | F | p-value |  |
| Bees |  |  |  |  |  |  |  |  |  |  |  |
| Brassicaceae | 1 | 0.125 | 1.667 | **0.018** |  |  | 1 | 0.717 | 3.2265 | **0.001** |  |
| Rosaceae | 1 | 0.141 | 1.879 | **0.011** |  |  | 1 | 0.272 | 1.2225 | 0.172 |  |
| Fabaceae | 1 | 0.134 | 1.790 | **0.013** |  |  | 1 | 0.656 | 2.9511 | **0.001** |  |
| Salicaceae | 1 | 0.115 | 1.542 | **0.044** |  |  | 1 | 0.389 | 1.7513 | **0.032** |  |
| Boraginaceae | 1 | 0.086 | 1.150 | 0.237 |  |  | 1 | 0.230 | 1.0373 | 0.367 |  |
| Apiaceae | 1 | 0.065 | 0.875 | 0.666 |  |  | 1 | 0.286 | 1.2887 | 0.204 |  |
| Asteraceae | 1 | 0.062 | 0.823 | 0.719 |  |  | 1 | 0.418 | 1.8832 | **0.030** |  |
| Ericaceae | 1 | 0.079 | 1.050 | 0.384 |  |  | 1 | 0.146 | 0.6564 | 0.771 |  |
| Residual | 83 | 6.209 |  |  |  |  | 56 | 12.440 |  |  |  |
| Honey |  |  |  |  |  |  |  |  |  |  |  |
| Brassicaceae | 1 | 0.253 | 2.3136 | **0.004** |  |  | 1 | 0.960 | 4.557 | **0.001** |  |
| Rosaceae | 1 | 0.495 | 4.5259 | **0.001** |  |  | 1 | 0.621 | 2.949 | **0.001** |  |
| Fabaceae | 1 | 0.352 | 3.2172 | **0.001** |  |  | 1 | 0.628 | 2.980 | **0.001** |  |
| Salicaceae | 1 | 0.315 | 2.8799 | **0.001** |  |  | 1 | 0.487 | 2.314 | **0.003** |  |
| Boraginaceae | 1 | 0.145 | 1.3222 | 0.113 |  |  | 1 | 0.221 | 1.052 | 0.385 |  |
| Apiaceae | 1 | 0.196 | 1.7956 | **0.033** |  |  | 1 | 0.355 | 1.686 | **0.050** |  |
| Asteraceae | 1 | 0.133 | 1.212 | 0.199 |  |  | 1 | 0.113 | 0.536 | 0.982 |  |
| Ericaceae | 1 | 0.114 | 1.0378 | 0.406 |  |  | 1 | 0.165 | 0.784 | 0.710 |  |
| Residual | 86 | 9.409 |  |  |  |  | 86 | 18.109 |  |  |  |


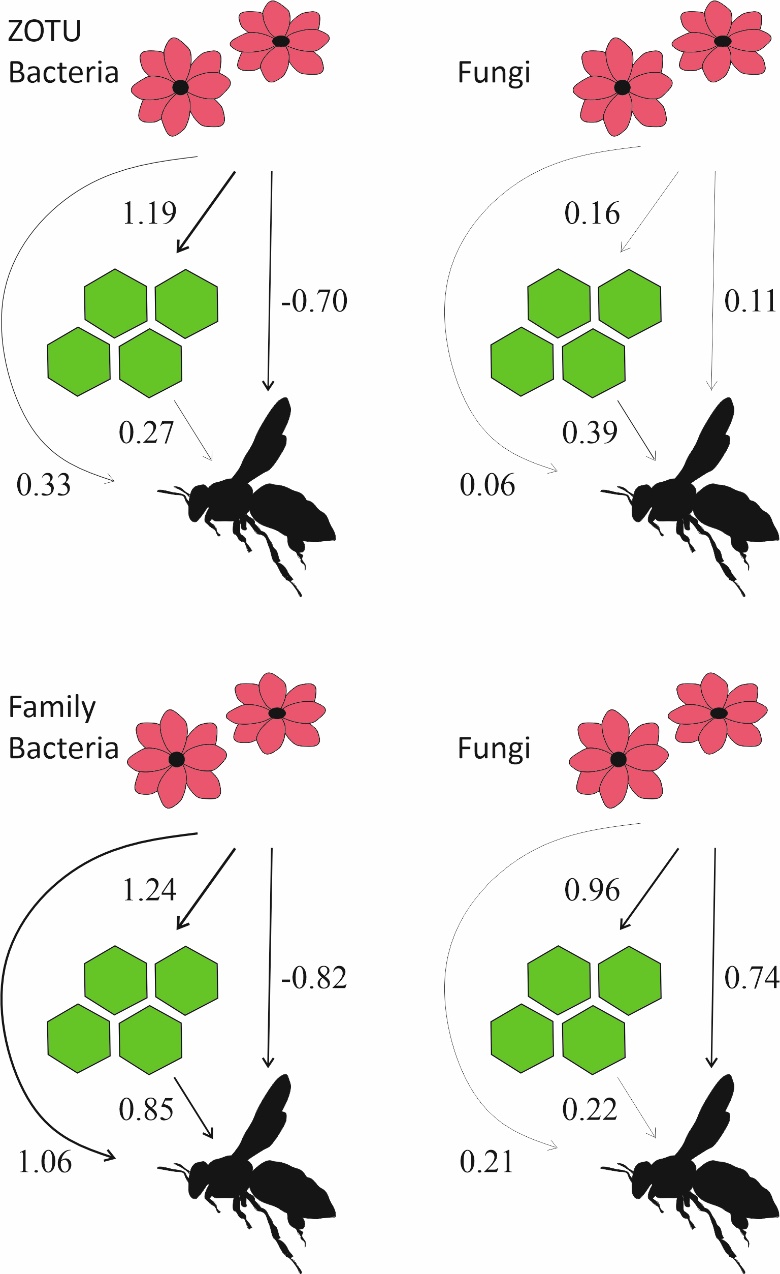


Figure S3. Pathways of the bacterial and fungal ZOTUs and families from flowers to honey and to bees. The panels with ZOTUs are identical to the ones shown in Fig. 3.

Table S11. Detailed results of the SEM pathway analyses for bacterial and fungal ZOTUs and families from flowers and honey to bees.

| ZOTU | Taxonomic group | | Bacteria |  |  |  |  |  | Fungi |  |  |  |  |  |
| --- | --- | --- | --- | --- | --- | --- | --- | --- | --- | --- | --- | --- | --- | --- |
|  | Number of observations | | 567 |  |  |  |  |  | 1072 |  |  |  |  |  |
|  | Regressions | | Estimate | Std. Error | z-value | P-value | Std. lv | Std. all | Estimate | Std. Error | z-value | P-value | Std. lv | Std. all |
|  |  | Honey Prop. ~ Flower Prop. (a) | **1.194** | 0.126 | 9.483 | **0.000** | 1.194 | 0.370 | **0.162** | 0.042 | 3.864 | **0.000** | 0.162 | 0.117 |
|  |  | BeeProp. ~ Honey Prop. (b) | **0.274** | 0.075 | 3.678 | **0.000** | 0.274 | 0.164 | **0.390** | 0.010 | 37.791 | **0.000** | 0.390 | 0.738 |
|  |  | BeeProp. ~ Flower Prop. | **-0.702** | 0.241 | -2.918 | **0.004** | -0.702 | -0.130 | **0.114** | 0.014 | 7.981 | **0.000** | 0.114 | 0.156 |
|  | Variances | |  |  |  |  |  |  |  |  |  |  |  |  |
|  |  | .Honey Proportion | 0.012 | 0.001 | 16.837 | **0.000** | 0.012 | 0.863 | 0.008 | 0.000 | 23.152 | **0.000** | 0.008 | 0.986 |
|  |  | .Bee Proportion | 0.037 | 0.002 | 16.837 | **0.000** | 0.037 | 0.972 | 0.001 | 0.000 | 23.152 | **0.000** | 0.001 | 0.404 |
|  | R-Square | |  |  |  |  |  |  |  |  |  |  |  |  |
|  |  | Honey Proportion | 0.137 |  |  |  |  |  | 0.014 |  |  |  |  |  |
|  |  | Bee Proportion | 0.028 |  |  |  |  |  | 0.596 |  |  |  |  |  |
|  | Defined Parameters | |  |  |  |  |  |  |  |  |  |  |  |  |
|  |  | ab | **0.327** | 0.095 | 3.429 | **0.001** | 0.327 | 0.061 | **0.063** | 0.016 | 3.844 | **0.000** | 0.063 | 0.087 |
| Family | Taxonomic group | | Bacteria |  |  |  |  |  | Fungi |  |  |  |  |  |
|  | Number of observations | | 79 |  |  |  |  |  | 93 |  |  |  |  |  |
|  | Regressions | | Estimate | Std. Error | z-value | P-value | Std. lv | Std. all | Estimate | Std. Error | z-value | P-value | Std. lv | Std. all |
|  |  | Honey Prop. ~ Flower Prop. (a) | **1.236** | 0.192 | 6.454 | **0.000** | 1.236 | 0.588 | **0.955** | 0.069 | 13.921 | **0.000** | 0.955 | 0.822 |
|  |  | BeeProp. ~ Honey Prop. (b) | **0.853** | 0.110 | 7.778 | **0.000** | 0.853 | 0.809 | **0.222** | 0.025 | 9.033 | **0.000** | 0.222 | 0.264 |
|  |  | BeeProp. ~ Flower Prop. | **-0.816** | 0.231 | -3.534 | **0.000** | -0.816 | -0.368 | **0.741** | 0.028 | 26.000 | **0.000** | 0.741 | 0.759 |
|  | Variances | |  |  |  |  |  |  |  |  |  |  |  |  |
|  |  | .Honey Proportion | 0.039 | 0.006 | 6.285 | **0.000** | 0.039 | 0.655 | 0.010 | 0.001 | 6.819 | **0.000** | 0.010 | 0.324 |
|  |  | .Bee Proportion | 0.037 | 0.006 | 6.285 | **0.000** | 0.037 | 0.560 | 0.001 | 0.000 | 6.819 | **0.000** | 0.001 | 0.026 |
|  | R-Square | |  |  |  |  |  |  |  |  |  |  |  |  |
|  |  | Honey Proportion | 0.345 |  |  |  |  |  | 0.676 |  |  |  |  |  |
|  |  | Bee Proportion | 0.440 |  |  |  |  |  | 0.974 |  |  |  |  |  |
|  | Defined Parameters | |  |  |  |  |  |  |  |  |  |  |  |  |
|  |  | ab | **1.055** | 0.212 | 4.967 | **0.000** | 1.055 | 0.475 | **0.212** | 0.028 | 7.578 | **0.000** | 0.212 | 0.217 |

**References for supplements**

1. Leponiemi M, Freitak D, Moreno-Torres M, et al (2023) Honeybees’ foraging choices for nectar and pollen revealed by DNA metabarcoding. Sci Rep in press: https://doi.org/10.1038/s41598-023-42102-4

2. Ribière C, Hegarty C, Stephenson H, et al (2019) Gut and Whole-Body Microbiota of the Honey Bee Separate Thriving and Non-thriving Hives. Microb Ecol 78:195–205. https://doi.org/10.1007/S00248-018-1287-9

3. Walters W, Hyde ER, Berg-lyons D, et al (2015) Transcribed Spacer Marker Gene Primers for Microbial Community Surveys. mSystems 1:e0009-15. https://doi.org/10.1128/mSystems.00009-15.Editor

4. Caporaso JG, Lauber CL, Walters WA, et al (2011) Global patterns of 16S rRNA diversity at a depth of millions of sequences per sample. Proc Natl Acad Sci U S A 108:4516–4522. https://doi.org/10.1073/PNAS.1000080107/SUPPL_FILE/PNAS.201000080SI.PDF

5. Toju H, Tanabe AS, Yamamoto S, Sato H (2012) High-Coverage ITS Primers for the DNA-Based Identification of Ascomycetes and Basidiomycetes in Environmental Samples. PLoS One 7:40863. https://doi.org/10.1371/journal.pone.0040863

6. Chen S, Yao H, Han J, et al (2010) Validation of the ITS2 region as a novel DNA barcode for identifying medicinal plant species. PLoS One 5:1–8. https://doi.org/10.1371/journal.pone.0008613

7. White T, Bruns T, Lee J, Taylor M (1990) Amplification and direct sequencing of fungal ribosomal RNA genes for phylogenetics. In: PCR protocols: a guide to methods and applications. Academic Press, pp 315–322

8. Vesterinen EJ, Puisto AIE, Blomberg AS, Lilley TM (2018) Table for five, please: Dietary partitioning in boreal bats. Ecol Evol 8:10914–10937. https://doi.org/10.1002/ece3.4559

9. Kaunisto KM, Roslin T, Forbes MR, et al (2020) Threats from the air: Damselfly predation on diverse prey taxa. J Anim Ecol 89:1365–1374. https://doi.org/10.1111/1365-2656.13184

10. Rognes T, Flouri T, Nichols B, et al (2016) VSEARCH: a versatile open source tool for metagenomics. PeerJ. https://doi.org/10.7717/peerj.2584

11. Cole JR, Wang Q, Cardenas E, et al (2008) The Ribosomal Database Project: improved alignments and new tools for rRNA analysis. Nucleic Acids Res 37:141–145. https://doi.org/10.1093/nar/gkn879

12. Nilsson RH, Larsson K-H, Taylor AFS, et al (2018) The UNITE database for molecular identification of fungi: handling dark taxa and parallel taxonomic classifications. Nucleic Acids Res 47:259–264. https://doi.org/10.1093/nar/gky1022

13. Banchi E, Ametrano CG, Greco S, et al (2020) PLANiTS: a curated sequence reference dataset for plant ITS DNA metabarcoding. 2020:155. https://doi.org/10.1093/database/baz155

14. Lee T, Alemseged Y, Mitchell A (2018) Dropping Hints: Estimating the diets of livestock in rangelands using DNA metabarcoding of faeces. Metabarcoding and Metagenomics 2:e22467. https://doi.org/10.3897/mbmg.2.22467

15. Alberdi A, Garin I, Aizpurua O, Aihartza J (2012) The foraging ecology of the Mountain Long-eared bat Plecotus macrobullaris revealed with DNA mini-barcodes. PLoS One 7:. https://doi.org/10.1371/journal.pone.0035692

16. Oksanen J, Blanchet FG, Friendly M, et al (2019) Package ‘vegan’ Title Community Ecology Package Version 2.5-6

17. Raymann K, Moran NA (2018) The role of the gut microbiome in health and disease of adult honey bee workers. Curr. Opin. Insect Sci. 26:97–104

18. Johnson KB, Stockwell VO, Burgett DM, et al (1993) Dispersal of Erwinia amylovora and Pseudomonas fluorescens by honey bees from hives to apple and pear blossoms. Phytopathology 83:478–484

19. Colda A, Bossaert S, Verreth C, et al (2021) Inoculation of pear flowers with Metschnikowia reukaufii and Acinetobacter nectaris enhances attraction of honeybees and hoverflies, but does not increase fruit and seed set. PLoS One 16:e0250203. https://doi.org/10.1371/JOURNAL.PONE.0250203

20. Evison SE, Jensen AB (2018) The biology and prevalence of fungal diseases in managed and wild bees. Curr. Opin. Insect Sci. 26:105–113

21. Foley K, Fazio G, Jensen AB, Hughes WOH (2014) The distribution of Aspergillus spp. opportunistic parasites in hives and their pathogenicity to honey bees. Vet Microbiol 169:203–210. https://doi.org/10.1016/J.VETMIC.2013.11.029
